# How reduced are nucleophilic gold complexes?†

**DOI:** 10.1039/d2dt01694j

**Published:** 2022-07-25

**Authors:** Isaac F. Leach, Diego Sorbelli, Leonardo Belpassi, Paola Belanzoni, Remco W. A. Havenith, Johannes E. M. N. Klein

**Affiliations:** Molecular Inorganic Chemistry, Stratingh Institute for Chemistry, University of Groningen Nijenborgh 4 9747 AG Groningen The Netherlands j.e.m.n.klein@rug.nl; Zernike Institute for Advanced Materials, University of Groningen Nijenborgh 4 9747 AG Groningen The Netherlands; Department of Chemistry, Biology and Biotechnology, University of Perugia Via Elce di Sotto 8 06123 Perugia Italy; CNR Institute of Chemical Science and Technologies “Giulio Natta” (CNR-SCITEC) Via Elce di Sotto 8 06123 Perugia Italy; Ghent Quantum Chemistry Group, Department of Chemistry, Ghent University 9000 Gent Belgium

## Abstract

Nucleophilic formal gold(-i) and gold(i) complexes are investigated *via* Intrinsic Bond Orbital analysis and Energy Decomposition Analysis, based on density functional theory calculations. The results indicate gold(0) centres engaging in electron-sharing bonding with Al- and B- based ligands. Multiconfigurational (CASSCF) calculations corroborate the findings, highlighting the gap between the electonic structures and the *oxidation state* formalism.

## Abstract 2

Nucleophilic formal gold(–i) and gold(i) complexes are investigated *via* Intrinsic Bond Orbital analysis and Energy Decomposition Analysis, based on density functional theory calculations.

## Graphical abstract

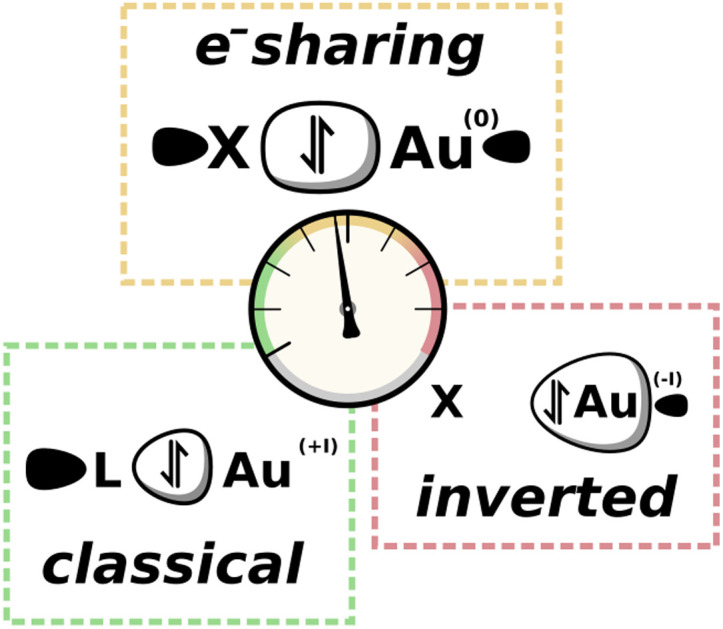

The remarkable two-coordinate gold complex 1 (Scheme 1), first synthesised by Aldridge and co-workers in 2019, is capable of inserting CO_2_ into its Au–Al bond, providing a nucleophilic source of gold.^1^ This “umpolung reactivity” is contrasted by gold's well studied electrophilicity,^2^ typical of gold(i) and gold(iii) species.^3–9^ Indeed, a wide variety of transformations are now known to be catalysed by gold complexes, including carbon bond-formation^10,11^ and oxygen atom transfer,^12–15^ so potential applications of 1 include waste valorisation and CO_2_ conversion.^16^ The aluminyl ligand [Al(NON)], where NON is 4,5-bis(2,6-diisopropylanilido)-2,7-di-*tert*-butyl-9,9-dimethylxanthene, is known to act as a strong σ-donor, and a polarised Au^*δ*−^–Al^*δ*+^ bond was reported,^1^ consistent with the (atomic) electronegativity (EN) difference: EN(Al, Au) = (1.61, 1.92) on the Allen scale.^17,18^ It follows that 1 is formally a gold(–i) species,^19^ which may at first seem unlikely but auride salts are stable in the solid state,^20–23^ in ionic liquids,^24^ and in liquid ammonia.^25^ Furthermore, theoretical work has proposed lithium aurides may be stable at very high (GPa) pressures, with the metal oxidation state (OS) tuneable down to gold(–iii).^26^ Gold's unique ability to accept electron density is well-known, ultimately due to relativistic lowering of the 6s orbital,^27^ making it more accessible for bonding. More generally, gold is now known experimentally to exist in a wide range of formal OSs (up to +V).^28–31^

**Scheme 1:**
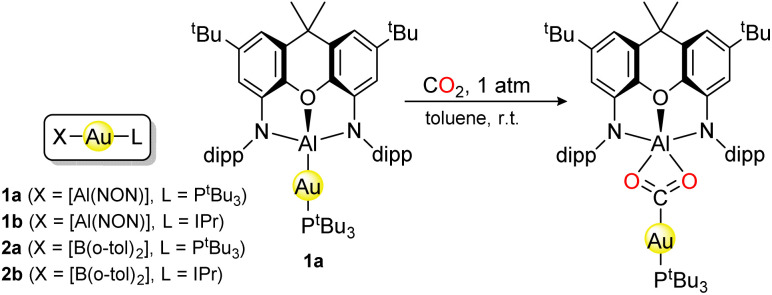
The nucleophilic complexes studied in this work (IPr = *N*,*N*′-bis(dipp)imidazole-2-ylidene, dipp = 2,6-^i^Pr_2_C_6_H_3_), and the CO_2_-insertion of 1a.

In 2021, Suzuki *et al.* reported 2, a diarylboryl analogue of the aluminyl complex 1 (Scheme 1), which also exhibits nucleophilic reactivity.^32^ In particular, 2b can perform insertion reactions with methyl-substituted carbodiimide, forming a species just like the CO_2_-insertion product of 1a. We might expect this similar reactivity to be reflected in equivalent OS assignment of the gold centres in 1 and 2. Curiously though, the EN of gold (1.92) lies between that of aluminium (1.61) and boron (2.05) on the Allen scale (the EN scale recommended by IUPAC).^19,33^1 and 2 are therefore formally gold(–i) and gold(i) complexes, respectively, a fact that seems hard to square with their chemical and structural parallels.

Typical two-coordinate gold complexes have the metal centre in the +I OS, with a formal 6s^0^5d^10^ configuration at the metal and a classical ligand-polarised dative covalent bond (Fig. 1a) for each of the two ligands. However, the strongly σ-donating [Al(NON)] ligand in 1 and EN(Au)>EN(Al), may lead us to expect more electron sharing (Fig. 1b) or even inverted (Fig. 1c) metal–ligand bonding scenarios. An inverted σ-bond was proposed,^1^ based on the observed nucleophilic reactivity and a calculated negative partial charge on the gold centre, but subsequent computational analysis by some of us points towards an electron-sharing covalent Au–Al bond as the source of nucleophilicity.^34–37^ The inverted scenario implies nucleophilic action of the gold centre, as the bonding pair of electrons is more closely associated to the metal, able of performing *e.g.* nucleophilic reduction of CO_2_ as in a proposed mechanism.^2^ Herein, we report a detailed computational analysis of the nucleophilic gold complexes 1 and 2 (Scheme 1), focused on the OS of the metal centre.

**Fig. 1:**
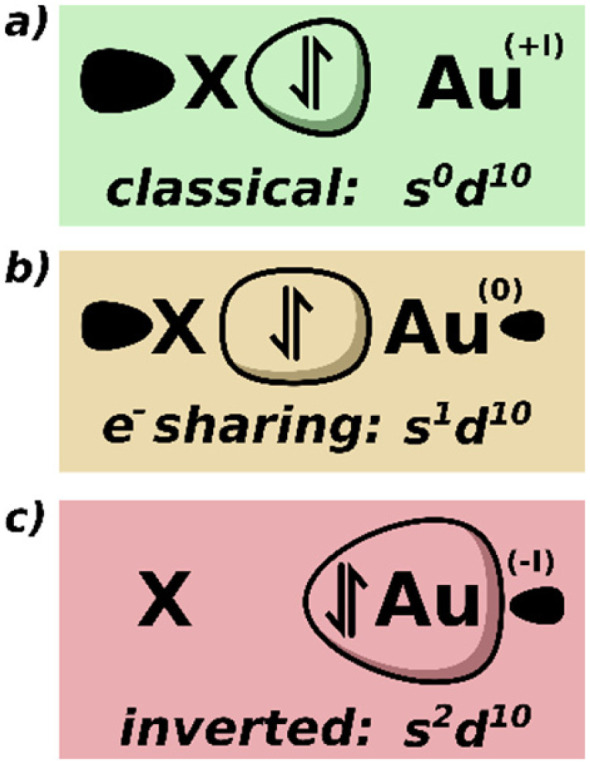
Possible bonding scenarios, their effective gold configurations and corresponding metal oxidation states for the Au–X bond, where X is [Al(NON)] (1) or [B(o-tol)_2_] (2) (see Scheme 1).

At the (B97-3c)^38^ optimized geometry, we performed single point calculations with the PBE0^39^/def2-TZVPP^40^ functional and basis set. Immediate insight into the nature of the Au–P and Al–Au σ-bonds of 1a can be gained *via* inspection of the Intrinsic Bonding Orbitals (IBOs)^41,42^ in Fig. 2A and B, respectively. The intrinsic atomic orbital (IAO)^41^ partial charge distribution of the Au–P bond, *q*_σ-IBO_(Au, P) = (0.19, 1.68), is typical for a dative covalent σ-interaction between gold and the l-type phosphine ligand.^43,44^ By contrast, the partial charge distribution of the Al–Au bond, *q*_σ-IBO_(Al, Au) = (1.17, 0.81), lies much closer towards the ideal electron-sharing bonding scenario, *q*_σ-IBO_(Au, X) = (1.00, 1.00). Similar results (Table S5†) were obtained using the B3LYP^45–48^-D3^49^(BJ)^50^/def2-SVP,^40^ PBEh-3c^51^ and GFN2-xTB^52^ methods, which performed well in our recent benchmark for efficient computation of geometries for gold complexes.^53^ This gold–aluminyl bond is significantly more electron-sharing than *e.g.*, gold–alkyl bonds in analogous complexes (Table S6†). The electron-sharing covalent bonding motif suggests an effective s^1^d^10^ configuration of gold(0) (Fig. 1b). The IBO analysis of the other X–Au–L bonds in 1 and 2 shows a maximum variation in *q*_σ-IBO_(Au) of only 6% (1a*vs.*2b in Table 1), consistent with the reduced ancillary ligand and aluminyl effects reported in ref. 33 and 34. Since the IBO localization procedure conveniently condenses the Au–X σ-interactions into a single orbital, examination of the IAO partial charge distributions provides a robust interpretation of the relevant bond polarity. This is contrasted by inspection of calculated atomic charges, which sum over all interactions and are not uniquely defined. In fact, one can always choose a partial charge definition and calculate either classical or inverted bond polarities in complexes 1 and 2 (Table S2,† see also ref. 32). This is just one example of the tenuous link between partial atomic charges and chemical OSs.^54–56^

**Fig. 2:**
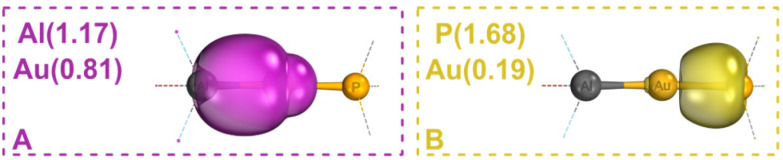
IBOs of 1a, calculated with PBE0/def2-TZVPP//B97-3c in ORCA 5.0.2,^57,58^ rendered in IboView.^41,42^ IAO partial charge distributions indicated in parenthesis.

**Table 1:** IAO partial charge distributions of the IBOs corresponding to the Au–X and Au–L σ-bonds in X–Au–L (Scheme 1)

Species	σ(IBO)^2^		σ(IBO)^2^	
	Au	X	Au	L
1a (X = Al, L = P)	0.81	1.17	0.19	1.68
1b (X = Al, L = C)	0.75	1.22	0.20	1.68
2a (X = B, L = P)	0.75	1.18	0.21	1.67
2b (X = B, L = C)	0.69	1.23	0.23	1.67

Energy decomposition analysis (EDA)^59–61^ is another approach to interpret DFT calculations that can complement the application of IBO analysis to probe bonding, oxidation states and metal configurations. EDA quantifies the various interaction energies between user-defined fragments and was previously applied in ref. 32 and 33 using [LAu] and X neutral radical fragments. Here we instead choose to fragment the molecule into the metal centre Au^*n*+^ and a united ligand fragment F^*n*−^. The charge, *n* = (−1, 0, 1), is varied to prepare the gold fragment in the (s^2^d^10^, s^1^d^10^, s^0^d^10^) configurations. We can judge which set of fragment orbitals are most similar to the combined molecule's orbitals by identifying the fragments with the smallest orbital interaction energy (Δ*E*_orb_).^62,63^ The results show that the s^1^d^10^ configuration is most favourable for 1 and 2 (Table 2), consistent with a gold(0) centre. The s^0^d^10^ configuration is in fairly close competition, particularly for 2b.

**Table 2:** Orbital interaction energies (Δ*E*_orb_) of all species in kcal mol^−1^, with the Au fragment prepared in the [Xe] 4f^14^5d^10^6s^n^ state with *n* = (0, 1, 2) corresponding to gold(i), gold(0) and gold(–i), respectively

Species	s^0^d^10^	s^1^d^10^	s^2^d^10^
1a (X = Al, L = P)	−166	−123	−273
1b (X = Al, L = C)	−173	−137	−294
2a (X = B, L = P)	−185	−151	−388
2b (X = B, L = C)	−196	−172	−370

To further probe the X–Au–L σ-bonding frameworks, CASSCF(4,4) calculations were performed at the optimized B97-3c geometries using Pipek-Mezey (PM) localization^65^ of the active space (see ESI for details†). Significantly populated active orbitals localized well onto each of the three atomic centres (A–C in Fig. 3). The most delocalized of the three, A, is 77% centred on its most contributing atom, Al. The σ-antibonding orbital D is spread over the three centres but is minimally occupied (0.09e). These fractional occupations numbers are obtained as a weighted sum of the integer occupation numbers in each configuration. In order to chemically interpret the results, we perform a Valence Bond (VB)-like reading of the CASSCF wavefunction, in the spirit of the work by Angeli, Malrieu, and co-workers.^66,67^ To find the portion of the wavefunction that corresponds to gold(0), we simply sum the weights of the configurations with the gold-centred orbital (Fig. 3B) singly occupied, here yielding a 51% contribution from gold(0). Additional contributions from gold(–i) and gold(i) are 33% and 15%, respectively. Very similar gold(0) contributions were found for the other complexes (53–54%, Table S1†). These results are consistent with both the IBOs and the EDA, which point towards the electron-sharing bonding of a s^1^d^10^ gold(0) centre (Fig. 1b). Validation of the VB-like reading of the CASSCF wavefunctions was obtained *via* VB-SCF calculations on two model complexes of 1a and 2a (see ESI† for more details). Although we note some variation in the minor gold(i) and gold(–i) contributions (Table S7†),^68^ the VB-SCF calculations similarly find the largest weight (>60%) for the gold(0) structure.

**Fig. 3:**
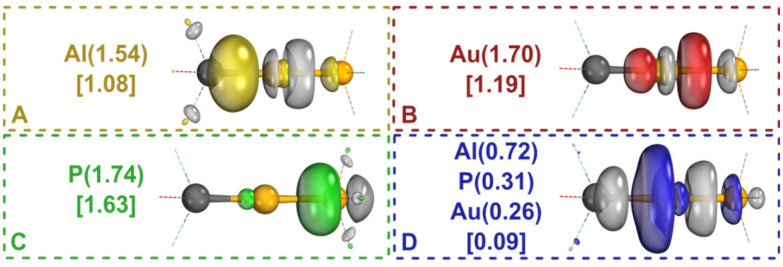
Active PM localized CASSCF(4,4) orbitals (A–D) of the Al–Au–P σ-bond in 1a, with their IAO partial charge distributions and occupation numbers shown in curved and square brackets, respectively. Calculated at the optimized B97-3c geometry in ORCA 5.0.2,^57,58^ rendered in IboView.^41,42^

A computational analysis of several nucleophilic gold complexes with aluminyl and boryl ligands is presented. We investigated the bonding scenario, using localized orbitals and energy decomposition analyses based on DFT calculations, and found that the electronic structures are consistent with 6s^1^5d^10^ gold(0) centres participating in electron-sharing covalent bonding with the Al- and B- based ligands. A valence-bond-like interpretation of CASSCF(4,4) calculations supports this assignment, indicating >50% gold(0) character for all species investigated, with some additional contributions from gold(–i) and gold(i). These results are in line with previous theoretical investigations,^34–37^ and lead us to echo recommendations to avoid using atomic partial charges for OS assignment.^54–56^ While the gold(0) OS assignment conveniently summarizes the electronic structures in chemical terms, it lies in stark contrast to the formal gold(–i) and gold(i) assignments of 1 and 2, highlighting the pitfalls associated with determining OSs based on atomic negativity differences alone. Similar conclusions have been reached by Salvador and co-workers when investigating transition metal complexes with Fischer and Schrock carbenes.^69–72^ Future efforts to pin down the elusive OS concept may benefit from EN definitions capable of accounting for the molecular environment, such as the charge-dependent EN concept introduced by Sanderson^73–75^ and pioneered by Pritchard^76–80^ – which provided the basis for more recent treatments of EN within conceptual DFT.^81,82^

## Conflicts of interest

There are no conflicts to declare.

## Supplementary Material

DT-052-D2DT01694J-s001
