# Supplementary material for: How reduced are nucleophilic gold complexes?
Source: Dalton Trans. 2022 Jul 25;52(1):11–5. doi: 10.1039/d2dt01694j (PMC9764324; doi:10.1039/d2dt01694j)
Supplement: DT-052-D2DT01694J-s001 [file DT-052-D2DT01694J-s001.pdf]

Electronic Supplementary Material (ESI) for Dalton Transactions.
This journal is © The Royal Society of Chemistry 2022

Supporting Information

for

How Reduced are Nucleophilic Gold Complexes?

Isaac F. Leach,[ab] Diego Sorbelli,[cd] Leonardo Belpassi,[d] Paola Belanzoni,[cd] Remco W. A. Havenith,[abe] and Johannes E. M. N. Klein[a,*]

[a]*Molecular Inorganic Chemistry, Stratingh Institute for Chemistry, University of Groningen, Nijenborgh 4, 9747 AG Groningen, The Netherlands. Email: j.e.m.n.klein@rug.nl*

[b]*Zernike Institute for Advanced Materials, University of Groningen, Nijenborgh 4, 9747 AG Groningen, The Netherlands.*

[c]*Department of Chemistry, Biology and Biotechnology, University of Perugia, Via Elce di Sotto, 8, 06123 Perugia, Italy.*

[d]*CNR Institute of Chemical Science and Technologies "Giulio Natta" (CNR-SCITEC), Via Elce di Sotto, 8, 06123 Perugia, Italy.*

[e]*Ghent Quantum Chemistry Group, Department of Inorganic and Physical Chemistry, Ghent University, 9000 Gent, Belgium.*

Table of Contents

Computational Details	S1
Examples inputs	S2
Energy Decomposition Analysis	S3
Au partial atomic charges	S4
IBOs of all species	S5
Method dependency of IBOs	
Comparison to alkyl gold complexes	
Valence bond calculations	S6
Cartesian Coordinates and Energies of Optimized Geometries	S7

Computational Details

All geometries were optimized in ORCA 5.0.2,[1, 2] using with the low-cost composite DFT method B97-3c from Grimme and co-workers[3] based on Becke's 1997 local exchange correlation functional[4] and uses the triple-zeta mTZVP basis set (based on the Ahlrichs basis set def2-TZVP)[5] and the corresponding effective core potential,[6, 7] to model relativistic effects by replacing the inner 60 electrons of gold. Dispersion effects are accounted for via the D3 model.[8, 9] Analytical frequencies were computed, and all structures were found to be well defined minima i.e. all calculated frequencies were positive. An energetic convergence criterion of 10^{-8} au was request via the *TightSCF* keyword. The default integration grid (*DefGrid2*) was used. Solvent effects were included via the conductor-like polarization continuum model (*CPCM*),[10] with toluene specified as the solvent.

Intrinsic bonding orbital analysis (IBO) was performed in IboView,[11-13] via additional single point calculations which were performed at the (B97-3c) optimized geometries with the PBE0[14]/def2-TZVPP[5] level of theory. The *RIJCOSX* approximation[15] was employed to speed up the integral evaluation, using Weigend's universal fitting basis set (*def2/J*). An energetic

convergence criterion of 10^{-8} au was requested via the *TightSCF* keyword. The default integration grid (*DefGrid2*) was used. Solvent effects were included via the conductor-like polarisation continuum model (*CPCM*),[10] with toluene specified as the solvent.

Structural depictions and orbital visualization of all species were produced with IboView[11-13] (source code available at http://www.iboview.org/). Orbital were localized with *exp 2*.

CASSCF(4,4) calculations were performed by starting with a RHF/def2-SVP calculation at the optimized B97-3c geometries, with separate Pipek-Mezey (PM) orbital localization of the occupied and virtual spaces in ORCA 5.0.2. The CASSCF active space was chosen by identifying σ-bonding and σ-antibonding orbitals with large coefficients on the Al, Au and P centres (see Figure S1 for an example). Once the CASSCF(4,4) calculation was converged, further PM localization of the active space only was performed in ORCA, followed by a final CAS-CI calculation. The PM localized active spaces of **1a**, **1b**, **2a** and **2b** and shown in Figure 3 and Table S1, respectively.

Examples inputs

**Geometry optimization**	*! B97-3c Opt Freq DefGrid2 TightSCF CPCM(Toluene) NoTrah SlowConv*
**Single point calculations**	*! PBE0 def2-TZVPP def2/J RIJCOSX DefGrid2 TightSCF CPCM(Toluene) NoTrah SlowConv*
**CASSCF(4,4) calculations**	*! def2-SVP def2/j def2-SVP/C RIJCOSX MoRead*<br>*%CASscf*<br>*TrafoStep RI*<br>*Nel 4*<br>*Norb 4*<br>*Mult 1*<br>*end*

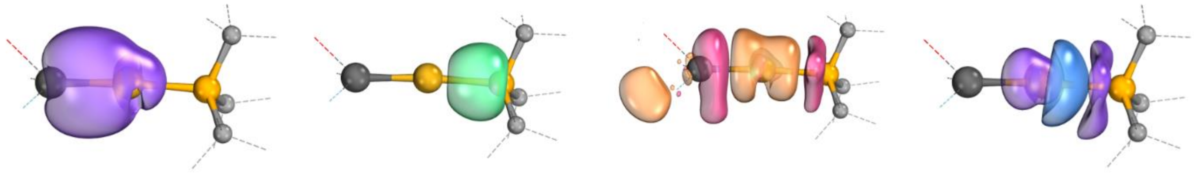

Figure S1: Localized PM orbitals of **1a** calculated with RHF, used as the initial active space in the subsequent CASSCF(4,4) calculation. Shown is the Al-Au-P(Me$_3$) moiety with hydrogens and all other atoms hidden. Rendered in IboView.

Table S1: PM localized active spaces of **1b**, **2a** and **2b**, calculated with CASSCF(4,4). The X-Au-L moieties are rendered in IboView with orbitals of arbitrary colours, all other atoms are hidden for clarity.

**Species**	**Active space** (Orbital partial charges) [Occupation number]				**Au OS (%)**		
					0	1-	1+
**1b**	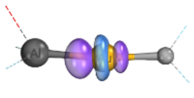<br>Al(0.16)<br>Au(1.64)<br>C(0.03)<br>[1.10]	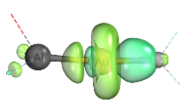<br>Al(0.32)<br>Au(0.77)<br>C(0.37)<br>[0.87]	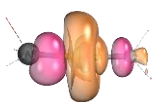<br>Al(0.73)<br>Au(0.56)<br>C(0.22)<br>[0.21]	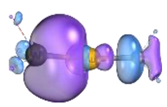<br>Al(1.15)<br>Au(0.34)<br>C(0.34)<br>[1.82]	53	29	18

**2a**	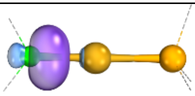 B(1.78) Au(0.18) [1.08]	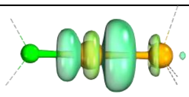 Au(1.76) P(0.08) [1.10]	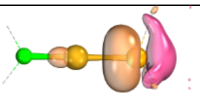 Au(0.14) P(1.68) [1.63]	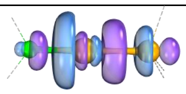 B(0.13) Au(0.19) P(0.37) [0.19]	54	27	18
**2b**	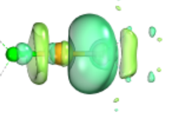 B(0.05) Au(0.31) C(0.26) [0.53]	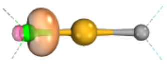 B(1.73) Au(0.13) [0.94]	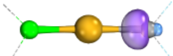 Au(0.10) C(1.82) [1.28]	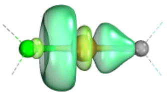 B(0.06) Au(1.58) C(0.05) [1.25]	53	35	10
**1a-VB**	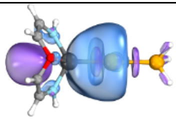 Al(1.45) Au(0.28) P(0.09) [0.92]	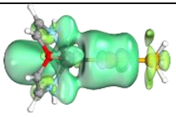 Al(0.24) Au(1.54) [1.08]	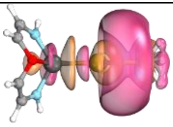 Al(0.18) Au(0.13) P(0.70) [0.00]	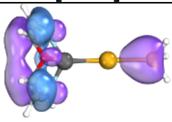 Al(0.02) P(0.54) [2.00]	50	29	21
					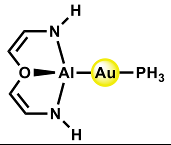		
**2a-VB**	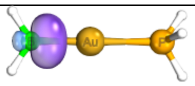 B(1.77) Au(0.18) P(0.02) [0.97]	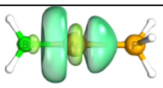 B(0.03) Au(1.91) P(0.05) [1.21]	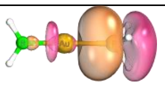 B(0.02) Au(0.05) P(1.62) [1.48]	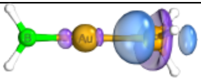 B(0.02) Au(0.07) P(1.25) [0.34]	55	32	7
					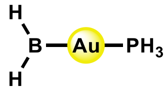		

Energy Decomposition Analysis

To further probe the electronic structure, and in particular the Au configuration, Morokuma-Ziegler Energy Decomposition Analysis (EDA)[16-18] was performed in the Amsterdam Density Functional (ADF) suite of the AMS2020 package.[19] These calculations were performed at the B97-3c optimized geometry from ORCA, and employed the PBE0 functional[14] in combination with the triple-ζ TZ2P basis set.[20] No frozen core approximation was made. Scalar relativistic effects were modelled *via* a ZORA Hamiltonian.[21-25] Numerical quality was defined with the *Good* keyword. For each EDA calculation only two fragments were defined: i) the metal centre, Au^{n+} and ii) the entire remaining ligand framework L^{n-}. The optimised coordinates were reoriented such that the Au-L bonds lay along the *xy* axes. The data in Table 2 are a summary of the results in Table S2.

Table S2: EDA of **1(a,b)** and **2(a,b)**, calculated with PBE0-ZORA/TZVP//B97-3c. Along with the instantaneous interaction energy (ΔE_{int}) the orbital interaction energy (ΔE_{orb}), the quasiclassical Coulomb interaction (ΔE_{elstat}) and repulsive Pauli exchange (ΔE_{Pauli}) terms are also given. All energies are in kcal mol^{-1}. For each species, the most favourable configuration (with the smallest ΔE_{orb}) is emphasized in bold.

Species	Au configuration	ΔE_{int}	ΔE_{orb}	ΔE_{elstat}	ΔE_{Pauli}
**1a**	[Xe]$4f^{14}5d^{10}6s^0$	-298.65	-165.98	-416.47	283.80
	**[Xe]$4f^{14}5d^{10}6s^1$**	-105.97	**-123.34**	-286.88	304.25
	[Xe]$4f^{14}5d^{10}6s^2$	-201.25	-273.03	-438.04	509.83
**1b**	[Xe]$4f^{14}5d^{10}6s^0$	-307.16	-173.13	-437.27	303.24
	**[Xe]$4f^{14}5d^{10}6s^1$**	-110.43	**-137.38**	-299.10	326.04
	[Xe]$4f^{14}5d^{10}6s^2$	-207.45	-294.03	-416.23	502.81

2a	[Xe]*4f^{14}5d^{10}6s^{0}*	-309.42	-185.34	-468.70	344.62
	**[Xe]*4f^{14}5d^{10}6s^{1}***	-108.33	**-151.00**	-307.67	350.35
	[Xe]*4f^{14}5d^{10}6s^{2}*	-207.81	-338.14	-450.51	580.83
**2b**	[Xe]*4f^{14}5d^{10}6s^{0}*	-319.50	-195.82	-488.31	364.64
	**[Xe]*4f^{14}5d^{10}6s^{1}***	-115.03	**-171.88**	-330.35	387.20
	[Xe]*4f^{14}5d^{10}6s^{2}*	-212.78	-370.26	-434.78	592.27

Au partial atomic charges

Partial atomic charges were calculated at the PBE0/def2-TZVPP level of theory, using various computational schemes. The Hirshfeld, Voronoi, Mulliken, Löwdin and Fuzzy partial atomic charges were calculated in MultiWFN 3.8.[26, 27] The IAO charges were calculated in IboView.[12]

Note: these calculations used pseudopotentials to replace the inner 60 electrons of gold,[6] and account for relativistic effects. When integrating electron density within an atomic basin (as in the fuzzy topological scheme), this causes a mismatch e.g. the integrated electron density is ~18 but the Au nuclear charge is set to 79, so the calculated net charge is ~61. To remedy this, the charge of Au in the ORCA molden file was manually changed to 19, as described in the MultiWFN manual. To verify the reliability of this procedure, an all-electron calculation was run for **1a**, using a ZORA Hamiltonian, the ZORA-def2-TZVPP basis set for all atoms expect gold (for which the SARC-ZORA-TZVPP basis set was used). A comparison of entries **1a** and **1a*** in Table S3 shows that Hirshfeld, Voronoi and Fuzzy atomic partial charges are less sensitive to a change in the level of theory, as compared to Muulliken and Löwdin charges.

Table S3: Partial atomic charges of **1** and **2** calculated within various computational schemes at the PBE0/def2-TZVPP//B97-3c level of theory. The more negative (less positive) atomic charge is highlighted in each case.

	**Au (X = Al, B) Partial Atomic Charge**					
	**Hirshfeld[28]**	**Voronoi[29]**	**Mulliken[30]**	**Löwdin[31]**	**IAO[12]**	**Becke[32]**
**1a**	-0.071<br>(0.241)	-0.125<br>(0.170)	-0.232<br>(0.106)	-0.003<br>(-0.291)	0.503<br>(0.084)	-0.965<br>(-0.046)
**1a***	-0.075<br>(0.241)	-0.129<br>(0.169)	-0.403<br>(0.342)	-0.620<br>(0.249)	/<br>/	-0.968<br>(-0.046)
**1b**	-0.053<br>(0.233)	-0.071<br>(0.150)	-0.222<br>(0.095)	0.125<br>(-0.299)	0.178<br>(0.472)	-1.254<br>(-0.076)
**2a**	0.036<br>(0.006)	0.006<br>(-0.077)	-0.172<br>(-0.130)	0.200<br>(-0.614)	0.182<br>(-0.169)	-1.237<br>(0.364)
**2b**	0.054<br>(0.000)	0.058<br>(-0.093)	-0.332<br>(-0.029)	0.331<br>(-0.620)	0.276<br>(-0.192)	-1.575<br>(0.361)

***** Calculated with PBE0-ZORA/def2-TZVPP (for a comparison of the relativistic effects modelled with pseudopotentials vs. all electron with a ZORA Hamiltonian).

IBOs of all species

In addition to the IBO analysis of complex **1a** (Figure 2) and the IAO partial charge distributions of all species (Table 1), the following plots of the key IBOs of **1a**, **1b, 2a** and **2b** are included here for completeness.

Table S4: (σ-IBO)^2 of the X-Au and Au-L bonds in complexes **1** and **2**, calculated with PBE0/def2-TZVPP//B97-3c. Orbital isosurfaces rendered in IboView[11, 12] using arbitrary colours to enclose 80% of their electron density.

Complex	X-Au	Au-L
**1a** (X=Al, L=P)	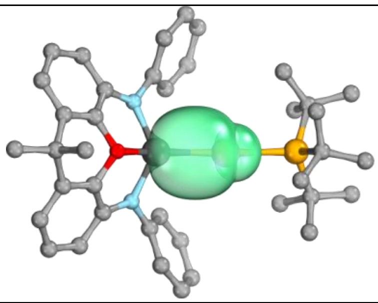	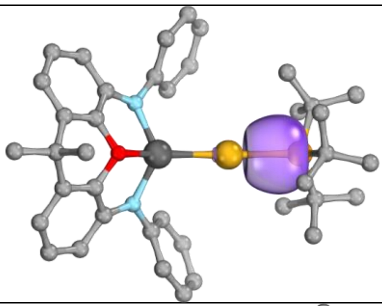
**1b** (X=Al, L=N)	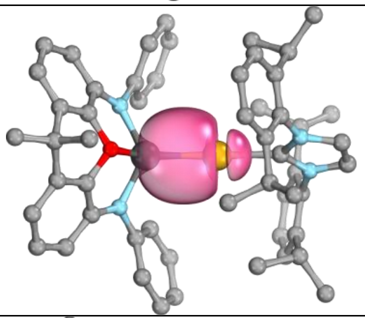	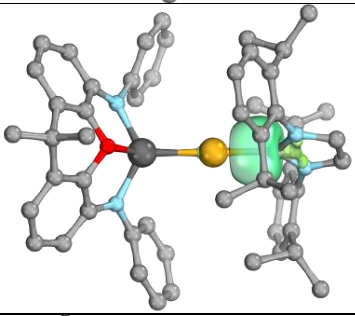
**2a** (X=B, L=P)	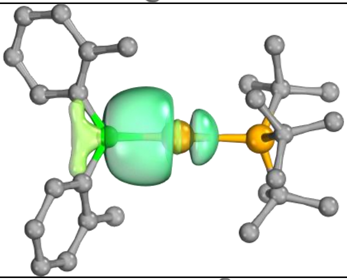	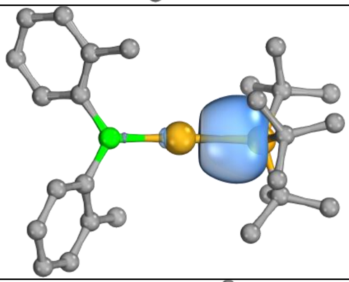
**2b** (X=B, L=N)	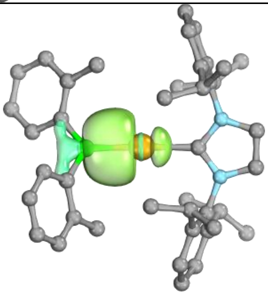	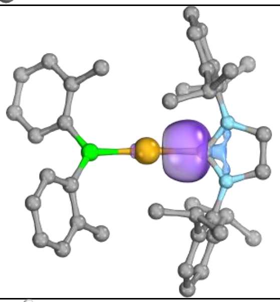
**1a-VB** (X=Al, L=P)	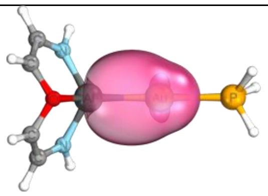 Al(1.04) Au(0.95)	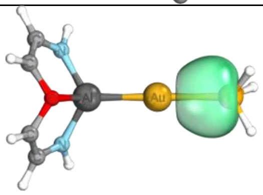 Au(0.10) P(1.81)
**2a-VB** (X=B, L=P) 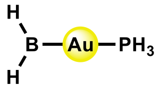	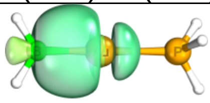 B(1.19) Au(0.81)	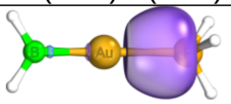 Au(0.16) P(1.77)

Method dependency of IBOs

Given the large method dependencies exhibited by partial atomic charges calculated with various schemes (Table S3), we sought to test the method dependence of the IAO partial charge distributions. To this end, we reoptimized the geometry of **1a** (starting from the B97-3c optimized geometry, *vide* supra) with a dispersion-corrected hybrid-GGA exchange-correlation functional paired with a spilt-valence basis set (B3LYP[33-36]-D3[8](BJ)[37]/def2-SVP)[5];

a composite DFT method featuring geometrical counterpoise corrections,[38] dispersion corrections[8, 37] and an increased percentage of HF exchange (PBEh-3c);[39] and a dispersion corrected[40] tight-binding DFT method (GFN2-xTB).[41] These methods performed well for gold complexes in our recent benchmark study.[42]

As for the other calculations, these optimizations were also performed in ORCA 5.0.2. Increased convergence thresholds we requested with the *TightOpt* keyword. The minima were verified *via* frequency analysis. An analytical Hessian was calculated for B3LYP-D3(BJ)/def2-SVP using the *Freq* keyword. Numerical Hessians were the requested for PBEh-3c and GFN2-xTB using the *NumFreq* keyword. For B3LYP-D3(BJ)/def2-SVP and PBEh-3c, the cPCM solvation model was used, with toluene as the solvent. For the GFN2-xTB calculation, the *xtb* executable (v6.4.1) was placed in the ORCA directory and was called using the *xTB2* keyword. The ALPB solvation modelled was used, with toluene specified as the solvent. All of these calculations used effective core potentials to replace the inner 60 electrons of gold.[6]

Additional single point calculations with PBE0/def2-TZVPP were performed at the optimized geometries to tease apart geometric and electronic effects in the method dependences. These calculations used the same settings as those performed at the optimized B97-3c geometry (*vide supra*). The doubly occupied σ-IBOs of the Al-Au and Au-P bonds calculated with these methods, and with the PBE0 single point calculations, are shown in Table S5. The crucial Al-Au bond polarity varies by just 0.11e. The largest variation (0.22e) is seen for the Au-P bond, calculated with GFN2-xTB vs. PBE0-def2-TZVPP//GFN2-xTB. With all tested methods, the IAO partial charge distributions of the Al-Au and Au-P bonds leave their respective characterization as electron-sharing and dative covalent, and therefore all conclusions regarding oxidation states, unchanged.

Table S5: $(\sigma\text{-IBO})^2$ and interatomic distances (*d*) of the Al-Au and Au-P bonds in complex **1a,** calculated with SP//GEOM (where SP = PBE0/def2-TZVPP, B3LYP-D3/def2-SVP, PBEh-3c or GFN2-xTB and GEOM = B3LYP-D3/def2-SVP, PBEh-3c or GFN2-xTB). Orbital isosurfaces rendered in IboView using arbitrary colours to enclose 80% of their electron density.

	**$(\sigma\text{-IBO})^2$**					
**GEOM =**	**(B3LYP-D3/def2-SVP,**		**PBEh-3c,**		**GFN2-xTB)**	
	| Al-Au	Au-P |	| Al-Au	Au-P |	| Al-Au	Au-P |
	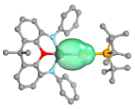 **Au(0.85) Al(1.13)**	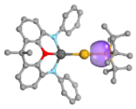 **Au(0.16) P(1.71)**	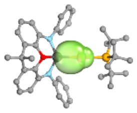 **Au(0.81) Al(1.18)**	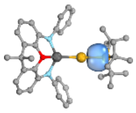 **Au(0.18) P(1.70)**	n/a	n/a
d (pm)	242	249	242	247	237	258
**SP=PBE0**	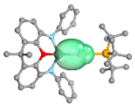 **Au(0.82) Al(1.16)**	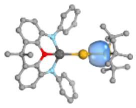 **Au(0.18) P(1.69)**	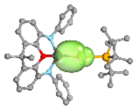 **Au(0.82) Al(1.17)**	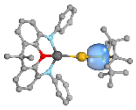 **Au(0.19) P(1.69)**	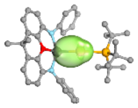 **Au(0.89) Al(1.09)**	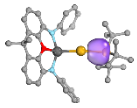 **Au(0.13) P(1.74)**

Comparison to alkyl gold complexes

To compare highly electron-sharing covalent nature of the Au-Al bond in **1** and **2**, we applied the same PBE0/def2-TZVPP//B97-3c level of theory (*vide supra*) to calculate the IAO partial charges in the X-Au-L alkyl gold complexes, where X = Me and L= (P^tBu_3, Me-Au-IPr) i.e. the same L ligands as in **1** and **2**. The gold-alkyl bonds are significantly less polarized than their gold-aluminyl and gold-boryl analogues (Table S6). If we choose some arbitrary threshold for 'ownership' of the bonding pair, say 70%, we might say that the Au-Me bonds lie close to the boundary between the electron-sharing and dative bonding regimes. Similar IAO partial charge distributions are found in other coinage metal carbon bonds e.g., $[Cu(CF_3)_4]^{1-}$.[43]

Table S6: $(\sigma\text{-IBO})^2$ of the Me-Au and Au-L bonds in the X-Au-L alkyl gold complexes, where X = Me and L= (P^tBu_3, IPr)**.** IPr = *N*,*N*'-bis(dipp)imidazole-2-ylidene, dipp = 2,6-$^iPr_2C_6H_3$. Calculated with PBE0/def2-TZVPP//B97-3c. Orbital isosurfaces rendered in IboView using arbitrary colours to enclose 80% of their electron density.

**L**	**(σ-IBO)²**	
	Me-Au	Au-L
**P^tBu_3**	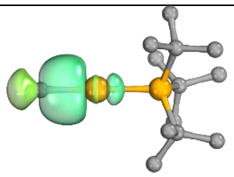 **Au(0.61) C(1.38)**	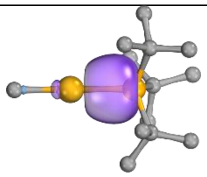 **Au(0.31) P(1.61)**
**Me-Au-IPr**	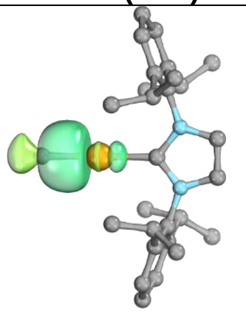 **Au(0.57) C(1.41)**	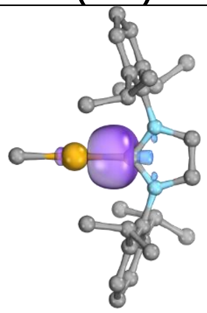 **Au(0.31) C(1.62)**

Valence bond calculations

Valence bond SCF calculations[44, 45] were performed with TURTLE,[46, 47] as implemented in GAMESS-UK.[48] For the Valence Bond calculations, the def2-SVP basis set[5] was used. The model molecules (**1a-VB** and **2a-VB**) were divided in two fragments, *viz.* the (PH_3)Au fragment and the $Al(C_4N_2H_6O)/BH_2$ fragment. Startup orbitals for the VB calculation were taken from a prior RHF calculation followed by a Pipek-Mezey[49] localization. The Au-M localized bond was used to form the two orbitals, that are used to form the spin coupled Au-M bond. Three structures were included in the calculation: **A** Au-M, **B** Au^+ M^-, and **C** Au^- M^+. The Gallup and Norbeck (GN)[50] and Chirgwin and Coulson (CC)[51] schemes are used to calculate the weights of the individual, non-orthogonal, VB structures. During the orbital optimization, the orbitals were kept localized on each fragment.

The final orbitals and their composition are depicted in Figure S2, and the weights of the individual structures are listed in Table S5.

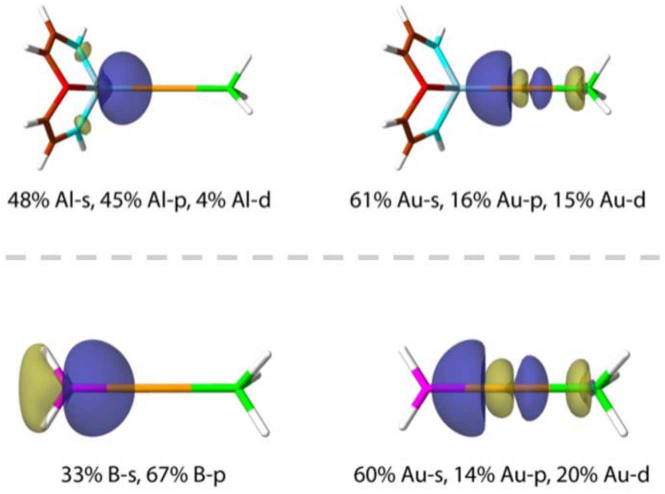

Figure S2: VB orbitals of the small model complexes **1a-VB** (top) and **2a-VB** (bottom).

Table S5: GN weights (CC weights) for the three structures.

Compound	Abbreviation	**A** Au-M	**B** $Au^+ M^-$	**C** $Au^- M^+$
$(PH_3)Au\text{-}Al(C_4N_2H_6O)$	**1a-VB**	0.620 (0.645)	0.033 (0.006)	0.374 (0.322)
$(PH_3)Au\text{-}BH_2$	**2a-VB**	0.681 (0.644)	0.242 (0.236)	0.077 (0.120)

The gold orbital in both compounds have a similar composition, and it has mainly s-character, with some p and d character. The orbital localized on Al is a sp hybrid, while the B orbital is more sp^2-like hybridized. For both models, the covalent structure has the highest weight. For Au-Al, the bond is polarized towards the Au atom, indicating a nucleophilic Au species. For Al-B, the polarity is reversed, with the $Au^+ B^-$ structure more important than the $Au^- B^+$ structure.

Cartesian Coordinates and Energies of Optimized Geometries

**1a** (X = [Al(NON)], L = P^{t}Bu$_3$)				(energies in Hartree)
C	3.13692693668037	-2.07055118534832	0.32757788632632	**E_{SCF}(B97-3c)**
C	3.73268716232558	-1.10164784106266	-0.48209395913235	**= -2419.486488402721**
C	5.01902497295678	-1.10871790872579	-0.96903056291391	
C	5.75941222847548	-2.26599862900996	-0.72177165116264	
C	5.18916691452319	-3.30243776571438	0.01274169270262	
C	3.90846144486486	-3.22127702188802	0.54449399311488	**E_{SCF}(PBE0/def2-TZVPP//B97-3c)**
C	5.51012897722653	0.15400920014500	-1.67854509045437	**= -2418.451047429955**
C	4.96026609618020	1.33871108601337	-0.88218904199947	
C	3.67509178250643	1.23862040854047	-0.40087141621526	
O	2.94663474657128	0.05739943957312	-0.64953019352750	
C	3.03123407451645	2.12523484379217	0.46467704110748	**$E_{CASSCF(4,4)}$**
C	3.75180757930392	3.29031073842015	0.76573033251145	**= -2405.295056048773**
C	5.02919526155845	3.46466237353921	0.24902154835286	
C	5.64624260193529	2.50870227980876	-0.55334168493653	
N	1.82058038204407	1.69050580048544	0.97168095729031	**Thermal correction* (B97-3c)**
C	0.91092221409208	2.56013297552737	1.58191991985275	**= 0.71324469**
Al	1.20645943773715	-0.02056858268897	0.36049533583880	
Au	-1.10444846577654	-0.03778565817711	-0.26955092975498	
P	-3.50308702662642	0.00289509075234	-0.72504894776648	
C	-3.81010449238163	0.07136557737410	-2.61327389315692	***1 atm, 298.15 K, 1M.**
C	-2.82830152306014	-0.89416872112607	-3.28787674478781	
C	7.02780231419447	0.19313348004775	-1.77226581571978	
C	4.92255564400657	0.19098215719678	-3.10290740201165	
N	1.91617949061719	-1.72265118452127	0.87257023911674	
C	1.06400906668995	-2.66895874688457	1.45378594362421	
C	-4.29689555312256	-1.57437192170792	0.01599503439668	
C	-3.62442265182861	-1.85187141324745	1.36534541984470	
C	-4.21711269182491	1.56571030825049	0.12046804804137	
C	-3.24130521998732	2.72379996360253	-0.11814691184728	
C	-5.81045275694486	-1.51265798707344	0.19406722285667	
C	-3.94312013153932	-2.76902405778289	-0.87000877841729	
C	-5.61609137259164	1.96466692750082	-0.33806246871130	
C	-4.22991957147919	1.34134533943458	1.63281345461966	
C	-5.23496769707346	-0.25481399013667	-3.04804716661784	
C	-3.43769083781634	1.46453408588588	-3.12040578496133	
H	3.52666183706032	-4.01974310518454	1.16284319812824	
H	6.77543864589858	-2.35624586288318	-1.07438215814695	
H	6.65681647194230	2.66946652739532	-0.89627138876820	
H	3.33471894522091	4.02657078137308	1.43594562952885	
H	7.35416738543763	1.09287955303778	-2.28855058322149	
H	7.39510625874914	-0.65413018260703	-2.34653483508367	
H	7.49472307869964	0.17150276127703	-0.78979798938876	
H	5.23718527451631	1.10071902197111	-3.61218355315560	
H	3.83616184270226	0.16654915549578	-3.08957396870034	
H	5.27581931055551	-0.66617208916135	-3.67427953913136	
H	5.77330123294331	-4.19344359556666	0.20213956854986	
H	5.57228121152281	4.36536956437284	0.50337782974330	
H	-4.24614777541663	-3.67827145096562	-0.34960291280749	
H	-4.45813122251226	-2.76064853365904	-1.82443713761829	
H	-2.87169090782604	-2.83527351652981	-1.04495210765258	
H	-3.99291149696110	-2.80615987560741	1.74501050106926	
H	-2.54542752681850	-1.93456097745831	1.26097514757708	
H	-3.83234418272931	-1.10229534547423	2.11704654925136	
H	-6.15964336805371	-2.47735613448666	0.56731693995305	
H	-6.11164062859930	-0.76296532013007	0.91896711942339	
H	-6.33354010465121	-1.31421796376133	-0.73649067890109	
H	-2.91801817504937	-0.77621416136384	-4.36900325553577	
H	-1.80086374122897	-0.66864915951374	-3.00902516880261	
H	-3.01718279003613	-1.93410056294663	-3.05818126376227	
H	-3.44881345822371	1.44411561724944	-4.21083424452515	
H	-4.13717805655114	2.23127534634131	-2.80679032276700	
H	-2.43598681670437	1.75057195657276	-2.80777671261544	
H	-5.31127218538434	-0.12863885919463	-4.12985470837382	
H	-5.51126448047183	-1.28103064463166	-2.82647267287936	
H	-5.97059471303162	0.39983847002764	-2.59113283850614	
H	-3.58857538623648	3.58737046236693	0.45054564563195	
H	-2.24161039525949	2.47926710887331	0.23327388817417	
H	-3.17092833356346	3.02510981658254	-1.15476339189126	
H	-5.95080080758492	2.81740895261344	0.25579797585822	
H	-5.63806299111446	2.27426297859328	-1.37845530733902	
H	-6.34202359592945	1.16846775591738	-0.20489646146775	
H	-4.47631403474628	2.28872442172684	2.11348913854685	
H	-4.97290418811613	0.61743858245064	1.94802402743807	
H	-3.25419519248307	1.03429718631358	2.00107263898006	
C	0.67239056719179	3.86219057180609	1.12464500990100	
C	-0.30413418306860	4.64968547288736	1.71135608217013	
C	-1.07938359534512	4.16202010316161	2.75768063713228	
C	-0.86372714926924	2.86721655647690	3.20759551988373	
C	0.12167726800234	2.07888403692383	2.63339918433156	
H	1.23558333351960	4.24243066511936	0.28460557914379	
H	-0.47275678721964	5.65061069812989	1.33492604329240	
H	-1.84373500279102	4.78044286289847	3.20862611935841	

H	-1.45887412823126	2.46632644148374	4.01774956291428	
H	0.30368910731421	1.08145723180836	3.01085543082498	
C	0.32852905879946	-2.30711399509432	2.58730228151599	
C	-0.60661292513403	-3.17099985076227	3.13678637285684	
C	-0.82325927052870	-4.42132897502724	2.57557786959001	
C	-0.09662929078778	-4.79154431360976	1.44895492720674	
C	0.83230241261356	-3.92915223136592	0.89059088961018	
H	0.51274450745062	-1.34455966540056	3.04605017028586	
H	-1.16506079622453	-2.86330616971228	4.01130046556244	
H	-1.54979641789324	-5.09754382044391	3.00564676413826	
H	-0.26434398712023	-5.75809261272191	0.99118616807386	
H	1.36052300180307	-4.21461814677865	-0.00813530010787	

**1a** (X = [Al(NON)], L = P^tBu_3)				(energies in Hartree)
C	3.15407694936855	-2.05070560580654	0.42495440484376	**E_{SCF}(PBEh-3c)<br>= -2415.701250696932**<br><br>**E_{SCF}(PBE0/def2-TZVPP//PBEh-3c)<br>= -2418.451414230496**<br><br>**Thermal correction* (PBEh-3c)<br>= 0.75101439**<br><br>***1 atm, 298.15 K, 1M.**
C	3.76099694270837	-1.10439720628618	-0.39631270073327	
C	5.03764335801657	-1.14493624746429	-0.90435883867503	
C	5.75948855966759	-2.30916902435599	-0.64756147737901	
C	5.17838541137859	-3.32058686618450	0.10950200588237	
C	3.90581725073854	-3.20785898966645	0.65569184219275	
C	5.53716872997928	0.09194637876823	-1.64796322423310	
C	5.00088551757243	1.29822089295681	-0.88000852597497	
C	3.72532546273850	1.21072554351034	-0.37441236048737	
O	2.99875769922987	0.04383570472802	-0.58343119601318	
C	3.08823349730347	2.12513679740576	0.45981877734207	
C	3.80689490309556	3.29838993436578	0.71457010760939	
C	5.07702284555731	3.45760074233847	0.17458643784630	
C	5.68791216848027	2.47790944609914	-0.60052057756582	
N	1.87480712377614	1.71420205733448	0.96511407706684	
C	0.96936304412385	2.62524060836499	1.51210760976439	
Al	1.24308023987625	0.00670773343044	0.38778563978545	
Au	-1.08795862692039	-0.01646527539534	-0.25821816015745	
P	-3.51252442080465	-0.00354930196097	-0.71110218280173	
C	-3.84638929765873	0.17753547445582	-2.57848053615672	
C	-2.85136790046573	-0.71001106987938	-3.34213424012064	
C	7.05899882346145	0.11565771188407	-1.73238266112193	
C	4.96439253257484	0.09800820089650	-3.07770870218892	
N	1.93311702284644	-1.68262295885481	0.94289645669692	
C	1.06720543305651	-2.62540218738907	1.50153033993794	
C	-4.27202010915092	-1.63444417318013	-0.08471889432530	
C	-3.61693866675361	-2.00000524770672	1.25625772810366	
C	-4.29445307841319	1.46311280306678	0.22078044585215	
C	-3.38202089351355	2.69044360969497	0.06776596992317	
C	-5.79223225298986	-1.61701958025029	0.08516791966627	
C	-3.89893670522997	-2.77429081230838	-1.03896619482871	
C	-5.70958982736149	1.83027265614018	-0.22856081563882	
C	-4.31945417937406	1.16269898993925	1.72377357743108	
C	-5.26847355124607	-0.16927657983041	-3.02212893895995	
C	-3.53442973337306	1.61188845696320	-3.01765319755455	
H	3.51740337788862	-3.99895821573713	1.28251533109325	
H	6.76960526109481	-2.42812229600139	-1.01334810618959	
H	6.69453753291600	2.63281504325896	-0.96239469445172	
H	3.39511427647040	4.06654320095149	1.35479994729959	
H	7.40086056795455	1.00083851069581	-2.26873132323840	
H	7.42684229174162	-0.74825228358069	-2.28590949165537	
H	7.52305083218516	0.11287226471170	-0.74578986308050	
H	5.29220153678005	0.99237423467522	-3.60957445242475	
H	3.87524414084444	0.08132924676277	-3.09118131761871	
H	5.31941856096052	-0.77456321411162	-3.62793686899545	
H	5.74742978160399	-4.22020679370871	0.30637111183771	
H	5.61963692315614	4.36915761118679	0.39017230075628	
H	-4.19004576312041	-3.71863365027929	-0.57236079728378	
H	-4.41303140335273	-2.72400906948157	-1.99644219064555	
H	-2.82419488359295	-2.82384004717281	-1.21958008209102	
H	-4.04111796526062	-2.94740365879368	1.59992368402863	
H	-2.54130785756306	-2.14886560011139	1.15958737649857	
H	-3.78146235876292	-1.27257906950835	2.04454441029792	
H	-6.12710345854659	-2.61564412569427	0.37851399294795	
H	-6.11944834460368	-0.93282024175889	0.86578187975115	
H	-6.31820960866051	-1.35622619945895	-0.83134712560613	
H	-3.00684229185555	-0.56081519338564	-4.41395121891551	
H	-1.81756142748022	-0.44087423953717	-3.12252189563598	
H	-2.96683210652831	-1.77128174480201	-3.14755928544607	
H	-3.56760763404317	1.64998963487778	-4.10931841316944	
H	-4.25455556346392	2.34259733547048	-2.65578501741182	
H	-2.53542571062429	1.92802530738111	-2.71391152353950	
H	-5.36396229004112	0.03181301340664	-4.09266457851501	
H	-5.50938330294543	-1.22053712401157	-2.87553912468957	
H	-6.02720999154512	0.42259561813969	-2.51383503377858	
H	-3.82425409116720	3.51915961696448	0.62727906742677	
H	-2.38964865411375	2.51889342872370	0.48614682654621	
H	-3.25889472746086	3.02691226757571	-0.95666830905333	
H	-6.09025692324889	2.62772912572467	0.41527826168242	
H	-5.74002351800019	2.20815214250158	-1.24877891093640	

H	-6.40662774321347	0.99737569210592	-0.15883421530983
H	-4.60896798696542	2.07664704685319	2.24824121511359
H	-5.04039132705856	0.39680262507954	2.00137349329292
H	-3.33718805556646	0.87432777396805	2.10087217113499
C	0.71618631772244	3.87526364057264	0.93635382919684
C	-0.24411079737754	4.71867881172536	1.46981814958597
C	-0.99146535463108	4.33410148416348	2.57656338733715
C	-0.76148380689489	3.08949171577730	3.14326372115762
C	0.21230920807985	2.24787456801616	2.62408129549479
H	1.26252677507927	4.18036851074951	0.05282533169836
H	-0.42052260844720	5.68052870854185	1.00493068831921
H	-1.74443447771504	4.99407216896078	2.98658352030235
H	-1.33398557221238	2.77110045695829	4.00527047680567
H	0.40321176646022	1.28959948665225	3.09366222179027
C	0.34698802860671	-2.29246909125353	2.65071722451234
C	-0.58795834762669	-3.16845249278178	3.18422921107178
C	-0.81544815192324	-4.40133409562640	2.59158336904060
C	-0.10427232202330	-4.74143342994675	1.44695030029297
C	0.81866822891459	-3.86447746771369	0.90164108106405
H	0.53531261060979	-1.34162250231342	3.13614969488255
H	-1.13479122528823	-2.88486235350835	4.07459212150530
H	-1.53882369900746	-5.08743443424301	3.01178390408689
H	-0.27947011619528	-5.69425747840002	0.96345660464538
H	1.33929917676356	-4.13353379603467	-0.00887527784404

1a (X = [Al(NON)], L = P^tBu_3) (energies in Hartree)

C	3.20711865271610	-2.08556542954660	0.35237839771260
C	3.83029518299099	-1.10886797204374	-0.44367543706216
C	5.13789596032445	-1.11474822170085	-0.90184800584556
C	5.87951175613349	-2.27724653188391	-0.62995691784241
C	5.28498211602997	-3.32061533520957	0.09390643375900
C	3.97969433041496	-3.24179071776337	0.59420664001698
C	5.63873011762181	0.15432122751393	-1.61555309691044
C	5.05695376263407	1.35048260051462	-0.83955251429629
C	3.75206521588948	1.23832913678137	-0.38596919058478
O	3.04927398348693	0.04564595922745	-0.63122349453120
C	3.06573262987776	2.13436259930610	0.45235663569320
C	3.76518252886819	3.32277759398078	0.75494607716859
C	5.06382421312841	3.50830525193669	0.26565394167701
C	5.72306651906042	2.54247144371623	-0.50773890896022
N	1.83948658573639	1.68582538669272	0.92172444534147
C	0.89497458517680	2.54464346690280	1.50594935125552
Al	1.26094193295218	-0.03834219737558	0.28924690734800
Au	-1.06529679999287	-0.06048694540377	-0.36264959107672
P	-3.52987165794534	0.00780313530625	-0.73481976383435
C	-3.94434794292056	0.08779775227099	-2.60938303905619
C	-2.99713928996042	-0.88829066843803	-3.34156841841937
C	7.16816636480848	0.20545744216958	-1.66947063650366
C	5.07938540369284	0.17262015352772	-3.06116382883549
N	1.95877952963870	-1.73874826146626	0.84694158985650
C	1.08365709865409	-2.68644601939564	1.40417160935596
C	-4.32081259571191	-1.56459906736024	0.03718705192485
C	-3.58830170858866	-1.86177014583363	1.36341847503766
C	-4.20315853745109	1.57620775883315	0.14988084889219
C	-3.22734674873653	2.73979116426013	-0.13274242171254
C	-5.83445609529010	-1.47364624467670	0.28517812133984
C	-4.02940564164494	-2.77198604302638	-0.87378526827429
C	-5.62895908666455	1.98351079427213	-0.25281610223538
C	-4.15493537674518	1.35065895397157	1.67271889052011
C	-5.40309302263085	-0.23465270177869	-2.96997195573754
C	-3.59440304374211	1.49048088642936	-3.14002005769314
H	3.57546223815657	-4.05301856876690	1.20034277357076
H	6.91347432635317	-2.36804450200145	-0.96308904192651
H	6.74918428529224	2.71405575753824	-0.83289149945517
H	3.31172456418887	4.07597710335991	1.39952748641278
H	7.50441882207780	1.11479901057178	-2.18866161014361
H	7.56139572492208	-0.65422164694470	-2.23144808496359
H	7.61113289807440	0.19527760360871	-0.66233869441803
H	5.39841027791124	1.09119340559128	-3.57813256256165
H	3.98034006571096	0.13741735355883	-3.06746496369338
H	5.45566397988942	-0.69676130845738	-3.62274646033312
H	5.87028674052050	-4.22058791489534	0.30145237083506
H	5.59096058010796	4.43155764054312	0.52111695592134
H	-4.32929644761629	-3.68706332112232	-0.33678163948904
H	-4.59132781007541	-2.74825614865875	-1.81576510111410
H	-2.95619145146636	-2.85935610754168	-1.09995281520391
H	-3.97170043415024	-2.81332577712007	1.76854945954777
H	-2.50658381778048	-1.98043945846102	1.20570458754640
H	-3.73615925527300	-1.09375316070040	2.12898491992688
H	-6.19003619028573	-2.44281705102566	0.67562414285942
H	-6.08995522166591	-0.71096213269020	1.03254085258313
H	-6.39871734903029	-1.25909370139069	-0.63207335314296
H	-3.14321459018107	-0.76843603317802	-4.42841083240419
H	-1.94384787412270	-0.66399592211828	-3.11268034916342

E_{SCF}(B3LYP-D3(BJ)/def2-SVP)
= -2417.719212809401

E_{SCF}(PBE0/def2-TZVPP// B3LYP-D3(BJ)/def2-SVP)
= -2418.444111209681

Thermal correction* (B3LYP-D3(BJ)/def2-SVP)
= 0.72132542

***1 atm, 298.15 K, 1M.**

H	-3.18130081539195	-1.93928837917584	-3.09782408904003	
H	-3.66804865867771	1.47076303473062	-4.24007890957228	
H	-4.28034030630389	2.26802567910136	-2.78207623420260	
H	-2.56438717199002	1.77788513453051	-2.88084675195724	
H	-5.53703917603639	-0.10834699527547	-4.05820078720012	
H	-5.67445644622080	-1.27082885068422	-2.72839115280422	
H	-6.11667248723685	0.43283341096767	-2.46910713097513	
H	-3.55334040183486	3.61669233523325	0.45057871565343	
H	-2.20428069179708	2.49365091529622	0.18783688388331	
H	-3.19588505418308	3.03665608007043	-1.18572852438470	
H	-5.93310852854340	2.85664802337187	0.34991963890790	
H	-5.69699128850352	2.28061132978253	-1.30777389622835	
H	-6.36098423021273	1.18541948850260	-0.07175859303394	
H	-4.37735263390345	2.30988421840178	2.16858807622902	
H	-4.89633433977430	0.62194758884880	2.02244584177907	
H	-3.15538770498268	1.03507691709481	2.00494101071696	
C	0.64103088110423	3.84756436818432	1.02812276245311	
C	-0.37096004825264	4.62768982213077	1.58942402052583	
C	-1.16746126341938	4.13029913695524	2.62782194761159	
C	-0.93541940730233	2.83504441548719	3.09927820446640	
C	0.08612351160946	2.05543756205566	2.55231136724836	
H	1.22703604322335	4.23696007849493	0.19397625439676	
H	-0.55072121127346	5.63249508565360	1.19690104770044	
H	-1.96237830774571	4.74370589782245	3.05850294464078	
H	-1.54867647153151	2.42597910405645	3.90653850941554	
H	0.27640782394317	1.05239316119038	2.94119990899835	
C	0.30505470538864	-2.32426740548171	2.52114938332786	
C	-0.65266733083319	-3.19687260750621	3.04327141612769	
C	-0.84833494446011	-4.45752260610153	2.47217441148004	
C	-0.07765270586487	-4.82955001416056	1.36349508734112	
C	0.87198121018655	-3.95689562641791	0.83020702771165	
H	0.46742138754961	-1.34751127904680	2.98325105558653	
H	-1.24753439196284	-2.88728480441502	3.90671963714920	
H	-1.59459286916619	-5.14220199160455	2.88229551006735	
H	-0.22725487497280	-5.80749801244348	0.89737057959798	
H	1.44120321600377	-4.24611654005981	-0.05520048229634	

**1a** (X = [Al(NON)], L = P^{t}Bu$_3$)				(energies in Hartree)
C	3.01740648434309	-2.23489712374852	0.01688022703639	**E$_{SCF}$(GFN2-xTB) = -126.091236242360**
C	3.71538641482607	-1.07128584534060	-0.35789898137160	
C	4.92778273450594	-1.04735071103624	-1.00669372309687	**E$_{SCF}$(PBE0/def2-TZVPP//GFN2-xTB) = -2418.420455195634**
C	5.46413113344579	-2.29160379631173	-1.34872557117078	
C	4.81346290960539	-3.45959111652176	-0.99126892779346	**Thermal correction* (GFN2-xTB) = 0.70061485**
C	3.61187478545332	-3.45498737894536	-0.30076116248936	
C	5.61708605623717	0.27150857939389	-1.32844426728803	***1 atm, 298.15 K, 1M.**
C	4.91021304533707	1.43152144182028	-0.64073118996057	
C	3.69697952282791	1.25147363707314	-0.01833237236193	
O	3.04347268378140	0.05026040931180	0.04861305811560	
C	2.97844030187637	2.25018391109356	0.66597671524594	
C	3.55744536701471	3.51702570042697	0.71841592576614	
C	4.76187017237475	3.73524187476424	0.06835388323953	
C	5.42924298551593	2.72852431768393	-0.60717445614572	
N	1.83738886479131	1.76522339652436	1.25433164443622	
C	0.84514865711541	2.57292078986260	1.77547567913403	
Al	1.32645228629193	-0.08389204046024	0.85707570306010	
Au	-0.78428404761651	0.04066455653990	-0.22307955657293	
P	-3.33099719097242	0.05799726875637	-0.61627374779056	
C	-3.79434581628510	-0.35531858498829	-2.43876754667037	
C	-2.84468359042488	-1.47550711036989	-2.89219841319848	
C	7.07550681567897	0.21014795096411	-0.84626099011052	
C	5.57362220127818	0.49644984313866	-2.84987331682046	
N	1.87856117998155	-1.95719204596011	0.72933019116214	
C	0.91927861317180	-2.90702030871723	1.02841472067747	
C	-4.12142783400522	-1.26081788744178	0.53702376016980	
C	-3.39093049596269	-1.18358878576912	1.88576234932635	
C	-4.04170211988752	1.78664149831873	-0.16356048500112	
C	-3.06278088248871	2.82906923162281	-0.72465422448581	
C	-5.63137667836566	-1.16815488231956	0.75779937666084	
C	-3.77815914302524	-2.64263277999816	-0.02980591980989	
C	-5.46434053736127	2.09273703223848	-0.63149380801736	
C	-3.96943416893252	1.94783503425968	1.35794003005586	
C	-5.24261161736498	-0.75329728778091	-2.72034840085113	
C	-3.43119498117083	0.86068973320926	-3.29850931107461	
H	3.15700651670121	-4.38567146121962	0.00217552254047	
H	6.39654226881147	-2.35360223208891	-1.88752398742434	
H	6.36138361533892	2.95543711828701	-1.10027719806192	
H	3.08928737442211	4.31525816964004	1.27372751749517	
H	7.59722128696000	1.13597066923479	-1.07231435577742	
H	7.60948613569509	-0.60233698480966	-1.33122679975360	
H	7.10633136607613	0.04998279121951	0.23007039374953	
H	6.06389160360985	1.43204320909125	-3.10960436988703	
H	4.54143786618476	0.54062906249903	-3.19395702140177	
H	6.07539667820228	-0.31729473504379	-3.36869440940453	
H	5.26177461129006	-4.40902965143616	-1.24953588490625	

H	5.19810797207592	4.72411315251562	0.09872554068616	
H	-3.99335570548193	-3.40340727041413	0.72044194378477	
H	-4.36674941478176	-2.88415854457169	-0.90937692797321	
H	-2.71610281019025	-2.70985692557425	-0.26362145405252	
H	-3.61569001672696	-2.07840376059844	2.46550370950068	
H	-2.31095197228956	-1.15105353164468	1.73366745346474	
H	-3.68351980372763	-0.32891445750590	2.48380809969779	
H	-5.95382246250777	-2.02776240379092	1.34665475820601	
H	-5.90651954473660	-0.27398818967730	1.31019325623423	
H	-6.17963799692671	-1.18127901983441	-0.17959333074713	
H	-2.85086665417320	-1.53584896719829	-3.98044462676663	
H	-1.82359410362974	-1.26374951385035	-2.57359992354122	
H	-3.12588713997656	-2.45144432338977	-2.51483804088259	
H	-3.45892890272561	0.57920189690839	-4.35156453792161	
H	-4.12983054056796	1.68121882435054	-3.16824302515156	
H	-2.42135801343754	1.20037321076610	-3.07233087865951	
H	-5.37325648414824	-0.86647060152052	-3.79737395432066	
H	-5.49880627626808	-1.70378469689320	-2.26065626225998	
H	-5.94354794969089	0.00197856793802	-2.37675147547310	
H	-3.29298398648276	3.80467145109375	-0.29724566664143	
H	-2.03958273326665	2.57948900761864	-0.44070493022881	
H	-3.11222282454624	2.92821388444127	-1.80247601315568	
H	-5.76761492044875	3.05877982161798	-0.22604840426582	
H	-5.53290638617314	2.15879263163915	-1.71362215299880	
H	-6.17237150362151	1.34685075948461	-0.28146774548842	
H	-4.12641055384058	2.99547230388861	1.61506389081140	
H	-4.73422152005703	1.37306174710207	1.87117608724347	
H	-2.98345868836951	1.66816595662472	1.72695678931018	
C	0.45454493805739	3.79684967933012	1.22263399223653	
C	-0.60771709696871	4.49898706958792	1.75730685552216	
C	-1.31785229979302	3.99919964035842	2.83730612096418	
C	-0.96435838507884	2.76955838838758	3.37132874258770	
C	0.09914616843096	2.06644352616017	2.84505630950517	
H	0.97006149227105	4.17839662158098	0.35315256651272	
H	-0.89148320256653	5.44642816773196	1.31905926708753	
H	-2.14337041113725	4.55785948871339	3.25430206952962	
H	-1.51990631307009	2.36177819823796	4.20508862959686	
H	0.39258107244292	1.10421030276416	3.25382015102502	
C	0.22289079340367	-2.76519181874309	2.23257949532438	
C	-0.80502306722036	-3.62176881966033	2.56854165616604	
C	-1.17269364639740	-4.63972420535946	1.70297028592920	
C	-0.51200236896994	-4.77295610546068	0.49163860302696	
C	0.51670045383441	-3.91756193105366	0.14946941208578	
H	0.52547682373275	-1.96363266320061	2.89956103380121	
H	-1.31977130106475	-3.49972478159419	3.51181890554171	
H	-1.97078669027829	-5.31918341701858	1.96567363750500	
H	-0.80917520823662	-5.54999877528495	-0.19980937556692	
H	0.99689575044351	-4.00728105166961	-0.81440883596473	

**1b** (X = [Al(NON)], L = IPr)				(energies in Hartree)
C	3.91089945797958	-2.41161679825403	0.14947345866795	**E_{SCF}(B97-3c)**
C	3.95137007420488	-1.17037442842628	-0.49224987868723	**= -2764.388293410669**
C	4.05944381232526	-1.02778150501743	-1.87705807459489	
C	4.13936133157717	-2.19435424658688	-2.63352150571970	
C	4.10396203051466	-3.43887817844097	-2.02807738534029	
C	3.98950968983498	-3.54611928628819	-0.65190236024308	**E_{SCF}(PBE0/def2-TZVPP//B97-3c)**
N	3.80597725449734	0.01058990517013	0.30841750262349	**= -2763.176633379318**
C	4.82591082142756	0.68853886805711	0.95488254673126	
C	4.24106860961707	1.71184952771212	1.62083558336124	
N	2.88557111752058	1.62524025378733	1.35445935207545	
C	2.60240622243384	0.57959427998628	0.54422584614400	**$E_{CASSCF(4,4)}$**
Au	0.66160644956764	-0.00175297965798	-0.08815061773357	**= -2746.339102317223**
Al	-1.60509680658079	-0.53112916751061	-0.61369389227327	
N	-2.46612138935301	0.60481795162565	-1.90303706192198	
C	-1.77388003337292	1.00447368625535	-3.05195293247632	**Thermal correction* (B97-3c)**
C	1.87046729987704	2.51327953868235	1.84335768334411	**= 0.89820432**
C	1.16748897368241	2.15540353016664	2.99693470894925	
C	0.16742030445174	3.02214739560725	3.42914208310848	
C	-0.12330376372608	4.18107157348293	2.72964328470699	***1 atm, 298.15 K, 1M.**
C	0.57516007666486	4.49532120962197	1.57563734802066	
C	1.58571563088168	3.66375761103142	1.10243133439558	
C	1.43208683968733	0.86773138262257	3.74065876470366	
C	1.91354208420120	1.13085780861091	5.16520074795166	
C	2.27655303322000	3.96237087825456	-0.20711354780565	
C	2.85672427746340	5.37216625149310	-0.25440383168241	
C	4.04202021077721	0.32398725733984	-2.55026385672020	
C	5.27475641601411	0.54425983389524	-3.42261213094574	
C	3.71677856515603	-2.53062270657815	1.64291151445877	
C	4.71106831421460	-3.49145344266192	2.28723081365027	
N	-2.23330540843733	-2.28549728153667	-0.12789285102116	
C	-3.37371916250962	-2.32147610545299	0.65599491102834	
C	-3.91596328965994	-1.07933767486717	0.99352874954546	
C	-5.12043183823973	-0.84004293143817	1.61522937724235	
C	-5.82911149608689	-1.96673783005517	2.03294781146207	

C	-5.31701793917196	-3.23410478644695	1.76906632341499	
C	-4.12223959839523	-3.42746705793021	1.08795885497137	
O	-3.19373673891497	0.01900335200368	0.48430825329488	
C	-4.03073008269250	0.93955728676446	-0.18132618016419	
C	-5.23248130049939	1.27603744043912	0.39624259351248	
C	-5.57141332771839	0.61753537420429	1.73406381716451	
C	-6.04382546737734	2.14350861113037	-0.33692803408250	
C	-5.62550227001044	2.57294314352099	-1.59382471830494	
C	-4.43624906737225	2.14512810553874	-2.16997328858821	
C	-3.59775122730774	1.26458663569075	-1.46874636014116	
C	-7.05339187966078	0.72565320712546	2.05740014539464	
C	-4.76506553684377	1.30318972772272	2.85460650367170	
C	-1.37347336756625	-3.37780472837869	-0.25612197316654	
C	0.20143391769114	-0.03677297418193	3.71989152422829	
C	1.32019946531498	3.70662613891314	-1.37103735727364	
C	2.75041536971992	0.51351272760996	-3.34068662453051	
C	2.27495863091607	-2.92633499815051	1.95547005779917	
H	-3.79772077042981	-4.42806374204586	0.84811494377552	
H	-6.77970393550703	-1.86594231944329	2.53378740297271	
H	-7.00028626429538	2.46273107635472	0.04779136149617	
H	-4.18344009128001	2.44732020376230	-3.17498316511898	
H	-7.35023459371834	1.76872848101623	2.13905452409143	
H	-7.26956673484611	0.25719369265733	3.01484849693744	
H	-7.66914509368216	0.25206111282481	1.29573022519729	
H	-5.05072514805095	2.35139209987960	2.93129627726277	
H	-3.69564603100633	1.25533204263759	2.66700790059153	
H	-4.96623728204986	0.81830581312932	3.80886680640543	
H	-5.88012653648745	-4.10331209272633	2.08266830279000	
H	-6.26284033420745	3.24098004522286	-2.15834828326598	
H	4.65360234122124	2.48351567611413	2.24353501979141	
H	5.85210232791195	0.37985568484071	0.88360091833034	
H	-0.40128195006850	2.77870647284978	4.31612027429185	
H	-0.90897742151440	4.83797070798484	3.07881716125141	
H	0.31727136563795	5.38930917408373	1.02483237091010	
H	4.21508773381033	-2.12662786926194	-3.71005043227780	
H	4.15425462394251	-4.33393154057908	-2.63400099868342	
H	3.93673337962557	-4.52496171637771	-0.19700991334742	
H	3.10753045451127	3.26827324433200	-0.31757561748585	
H	2.22608590756919	0.33463448784192	3.22181278943355	
H	4.05755657743360	1.08771367193390	-1.77489700127225	
H	3.88760435873601	-1.55005457011984	2.08422227671668	
H	2.70651767662718	1.51245415051556	-3.77202337744889	
H	2.67318548512237	-0.20491931285872	-4.15573836017666	
H	1.87654460881020	0.38628941940830	-2.70319005060748	
H	5.27050325588071	1.55476296687209	-3.83017923859255	
H	6.19295619663514	0.41385389840027	-2.85069817266936	
H	5.30448121973873	-0.14791724834688	-4.26317218807408	
H	4.60068377236525	-3.47141837465594	3.37091461380544	
H	4.54917031462052	-4.51845573195194	1.96332506085430	
H	5.73882040778718	-3.22213059379943	2.04618624688937	
H	2.11141166993041	-2.95436024755802	3.03268488468432	
H	1.57070772942216	-2.21792649516779	1.52158448803046	
H	2.04255060419593	-3.90836478579014	1.54953142487387	
H	2.15158238814818	0.19133427100645	5.66331049046904	
H	2.80723281975208	1.75408885006746	5.17250181182549	
H	1.15045963950591	1.63478349655695	5.75727521720451	
H	0.42977380662240	-0.99576713414953	4.18330137013998	
H	-0.63196144268159	0.40758156826352	4.26301775697016	
H	-0.12233922283976	-0.22382988435204	2.69646352856682	
H	1.82947518657066	3.84472653235581	-2.32430185639157	
H	0.92896063683117	2.69088633544943	-1.34337625671094	
H	0.47265865395149	4.39040232458524	-1.34006328621613	
H	3.41444494345986	5.51681984989287	-1.17908235947954	
H	2.07707449991669	6.13189382471343	-0.22205629385488	
H	3.53308713923508	5.55157120560101	0.58062563134570	
C	-1.23319893306924	0.02428360553019	-3.89031163451508	
C	-0.46557818455717	0.37126359307068	-4.99148555905638	
C	-0.22782869461116	1.70537216266414	-5.29008842270259	
C	-0.75773688376351	2.68798831655766	-4.46106779005171	
C	-1.51678816476821	2.34657462760482	-3.35458759813339	
H	-1.44439579054805	-1.01651672899154	-3.68235417219594	
H	-0.05724610565610	-0.40791405840511	-5.62186288530413	
H	0.36930516482632	1.97711459432500	-6.15014222265770	
H	-0.56392566406457	3.73270744984941	-4.66842978635751	
H	-1.88515407604194	3.11836312647529	-2.69406237006033	
C	-1.16328616025962	-4.32632677736124	0.75430013653874	
C	-0.23938096671879	-5.34483944645193	0.58874705535378	
C	0.52085635330026	-5.43933448044252	-0.57071299942132	
C	0.34224211908552	-4.48992584711482	-1.56745361743644	
C	-0.59702760082421	-3.48312872958427	-1.41885564219592	
H	-1.70062558782105	-4.24359047686748	1.68704289673274	
H	-0.09712674772351	-6.06097851985380	1.38826678970379	
H	1.24701715833657	-6.23232592562251	-0.68896803115278	
H	0.93151713096413	-4.53425344357092	-2.47373566567068	
H	-0.74505452888644	-2.76913414998626	-2.21822423985441	

**2a** (X = [B(o-tol)$_2$], L = P^{t}Bu$_3$)				(energies in Hartree)
B	0.03119944253388	0.02669515022817	-2.08389180299337	**E_{SCF}(B97-3c)<br>= -1517.227844113290<br><br>E_{SCF}(PBE0/def2-TZVPP//B97-3c)<br>= -1516.438379805822<br><br>$E_{CASSCF(4,4)}$<br>= -1507.814068277810<br><br>Thermal correction* (B97-3c)<br>= 0.54316597<br><br>*1 atm, 298.15 K, 1M.**
Au	0.06255373657645	0.03951446858255	-0.00318030298050	
C	0.63917708215237	2.53312203633110	-2.69828705606245	
C	0.66568228022691	1.14455658228228	-2.96775828424459	
C	1.41274630955167	0.70761629540729	-4.07443645627883	
C	2.15328637142696	1.57824458206306	-4.85918435249304	
C	2.12130579147430	2.93566328791227	-4.57891144400191	
C	1.35731728965069	3.40045073942398	-3.51555564973422	
P	0.06312218769904	0.01342722283748	2.43719226853472	
C	0.03269323109962	-1.80653943117702	3.03695259129896	
C	-0.17723653334202	3.09486203063275	-1.57635009835056	
C	-0.67580511123087	-1.15930403364265	-2.81362188127728	
C	-0.40244154482420	-2.52854377928547	-2.60369649255782	
C	-1.20126108702923	-3.48802612113582	-3.21834731350058	
C	-2.27244234419936	-3.13156520265038	-4.02766759345431	
C	-2.54016742778433	-1.78970004028325	-4.25739267418633	
C	-1.73346248841664	-0.82478305816688	-3.67339885380442	
C	0.77220085680939	-2.95380900566530	-1.77948654140690	
C	1.65100742072077	0.88575921100518	3.05808057172493	
C	-1.48655169480180	0.94051765100977	3.07495242392875	
H	1.32200189145768	4.46463897465751	-3.31496529582691	
H	2.67860980572594	3.63479935287038	-5.18946154732075	
H	2.73238138102929	1.20359250452999	-5.69359228707124	
H	1.41594628941993	-0.34882802493669	-4.31306535086673	
H	-3.35993688862702	-1.49709086432244	-4.90129975967578	
H	-2.88385454786346	-3.89882422025064	-4.48534295014957	
H	-0.98110170350247	-4.53738134242107	-3.06013626809343	
H	-1.93322379673628	0.22145605536946	-3.87435709536672	
H	-1.24040718209571	2.90333125282304	-1.71933393145065	
H	0.08834975915937	2.62036840330041	-0.62994361267654	
H	-0.03804765937502	4.16993119164934	-1.48138691029152	
H	0.72910565876113	-4.01194805018808	-1.52876528187194	
H	0.82646722709248	-2.37153146809980	-0.85707957324167	
H	1.71000693354301	-2.77374348161956	-2.30746136582645	
C	-0.32973100604450	-1.99701176942318	4.50658878257942	
C	-0.95874119131883	-2.58155857091083	2.16041915836535	
C	1.40267747391080	-2.43178898650576	2.77245336425768	
C	-1.43563122905109	1.34252961587716	4.54548815569040	
C	-1.68954495953064	2.18490259107797	2.20299918035778	
C	-2.71879906208014	0.06703465300027	2.83708702205660	
C	2.00298251808890	0.62689808107511	4.51932703613356	
C	2.81508039156516	0.44562699543220	2.16140222263007	
C	1.50702166311031	2.39231368956365	2.83743980552779	
H	3.69607303787909	1.03035202975469	2.43047919383206	
H	3.07474745163293	-0.59919118845986	2.26864333688593	
H	2.59575152542095	0.63242029910347	1.11196492078244	
H	2.47408452822256	2.85706967280373	3.03318013759416	
H	1.23642924541499	2.62441698359352	1.81004581976453	
H	0.78942988289051	2.85593314606778	3.50526324367710	
H	2.88456820111518	1.21613456473029	4.77928379758953	
H	1.20683754820386	0.91394628730521	5.19926697445789	
H	2.25035420671414	-0.41327245959934	4.70611317036642	
H	1.32489018289052	-3.50548739821835	2.94712656504290	
H	1.71856919592311	-2.29023152316182	1.74142548620471	
H	2.17574716684300	-2.05625606713895	3.43370992172262	
H	-0.87476810459810	-3.64191086456424	2.40306061291741	
H	-1.98964819914546	-2.29486249228874	2.31876937957686	
H	-0.73407892038963	-2.45982281738053	1.10363194711932	
H	-0.26003654723983	-3.05850966336162	4.75231180570417	
H	0.33871837005329	-1.46374228219555	5.17508371374300	
H	-1.34671911404921	-1.68657421922641	4.72393443492102	
H	-2.39071985825081	1.79454068588625	4.82004090937864	
H	-1.27529860765412	0.49434842302235	5.20350457246322	
H	-0.66464874439537	2.07926066698377	4.74794083146234	
H	-2.61319849188540	2.67459610841615	2.51509488658788	
H	-0.89211223231021	2.91092051745399	2.28522810411001	
H	-1.78973448418831	1.91440952139459	1.15435727623632	
H	-3.60565339021182	0.67366750532359	3.02426454509532	
H	-2.77207678282451	-0.28082880513380	1.80811753916328	
H	-2.77144260099381	-0.78790179936771	3.50213231757194	

**2b** (X = [B(o-tol)$_2$], L = IPr)				(energies in Hartree)
C	1.79703188590159	-2.99201193458970	0.98726702975410	**E_{SCF}(B97-3c)<br>= -1862.134268302139<br><br>E_{SCF}(PBE0/def2-TZVPP//B97-3c)<br>= -1861.166407763792**
C	2.22203566531718	-2.35574222385042	-0.18177474707697	
C	2.16911058908011	-2.95870569359729	-1.44148601875683	
C	1.70866352464248	-4.27042888420600	-1.50445535339871	
C	1.30730306804984	-4.93856054352650	-0.35954153906860	
C	1.34229589847583	-4.30289408569829	0.86926862904005	
N	2.70468534695922	-1.00833145264408	-0.09384637552877	
C	1.88768332374029	0.07095791729419	-0.09409081537367	
N	2.73448747482945	1.12163018353164	0.01493977662236	
C	4.05431485830963	0.70942157024691	0.08044573541990	

C	4.03516628542758	-0.64194101879128	0.01311266588811	**$E_{CASSCF(4,4)}$ = -1848.863824963147**<br><br>**Thermal correction* (B97-3c) = 0.72767547**<br><br>***1 atm, 298.15 K, 1M.**
Au	-0.22072708887097	0.07044335833261	-0.10615349973978	
B	-2.28690082830446	-0.00531636249165	0.02059880436937	
C	-2.91370678818629	-1.22963689996273	0.76495266640262	
C	-3.67576723927431	-0.95461562542601	1.90923140356607	
C	-4.12322593548695	-1.96077931167124	2.75381745149675	
C	-3.85583663596236	-3.28464864754548	2.43807244758795	
C	-3.14355041340319	-3.58326099953850	1.28263578269190	
C	-2.66044936348324	-2.58185730307089	0.44740627998832	
C	-1.89475783512508	-2.94102621607160	-0.78687165096418	
C	2.28286471449706	2.47907918365212	0.10795768286124	
C	2.05709892886416	3.01404927070640	1.37939683934063	
C	1.59844090348493	4.32614539954489	1.44854131602619	
C	1.37352612442010	5.06295240552350	0.29770480561826	
C	1.59195784420846	4.49909492376795	-0.94745182518345	
C	2.04845608608458	3.18968269543871	-1.07128968327953	
C	-3.24991013956121	1.08377296857115	-0.54576512204020	
C	-3.02532109550448	2.48034891742452	-0.51075317590375	
C	-3.89755453912795	3.32793383912664	-1.18693585082929	
C	-4.97527194382657	2.83678415722735	-1.91369980899417	
C	-5.21455101012188	1.47144777545998	-1.94798789840989	
C	-4.37244504881026	0.61884695137761	-1.25002575082972	
C	-1.89463626072720	3.06862131379930	0.27371907834396	
H	4.86656217694589	1.40604967296663	0.17161738953821	
H	4.82785901460028	-1.36640965634321	0.02478866649611	
H	-2.95457723624715	-4.61955573469119	1.02723437559118	
H	-4.21065444602807	-4.08311711589669	3.07744381685141	
H	-4.69401237565623	-1.71380217596847	3.64024514289767	
H	-3.90648336387558	0.07720024028879	2.14939216780362	
H	-6.06049766185608	1.07634125373837	-2.49617609301645	
H	-5.62933129966830	3.52123398904306	-2.43914251915847	
H	-3.72941751885863	4.39772684062310	-1.14736902176341	
H	-4.57664522543908	-0.44512998009548	-1.25578493472809	
H	-2.38964686752547	-2.57648136038616	-1.68719875240901	
H	-0.90739292916395	-2.47534994690132	-0.77160211424205	
H	-1.76458190241033	-4.01663604699358	-0.88227627961120	
H	-1.83205681683927	4.14613981124750	0.13803620915073	
H	-0.94105760019227	2.62625681793288	-0.01867399801960	
H	-2.00175868653018	2.86935754407956	1.33968272832286	
C	2.23256166960906	2.19323195764129	2.63596577100004	
H	1.39962568222957	4.77120898428345	2.41347326162533	
H	1.01124486172658	6.07976574557184	0.37189315096774	
H	1.38941173788915	5.07842948814707	-1.83761427953180	
C	2.22029222463507	2.56508219253463	-2.43560101523031	
C	2.50422941364268	-2.19731930150526	-2.70290212352644	
H	1.64609949282492	-4.76850291067981	-2.46174320110407	
H	0.94736071881012	-5.95657467691007	-0.42864108841291	
H	1.00340417861642	-4.82872669196578	1.75105136944139	
C	1.80131390658897	-2.30831407065602	2.33428606155227	
C	0.86072280540386	2.36833879072013	-3.10422347854593	
C	3.17372977950858	3.36583169475072	-3.31804655207557	
H	2.65656626653090	1.57702236415789	-2.30137355427766	
C	0.87007408622941	1.79317428195689	3.20089653631359	
H	2.74875108045760	1.27265435072172	2.36965280504333	
C	3.08942281477406	2.90071301828439	3.68114876702424	
C	1.21446025571996	-1.78750779872146	-3.41442699600890	
H	3.01819401841997	-1.28053291034266	-2.41947586219936	
C	3.43750778850098	-2.96838419382488	-3.62950916641929	
C	0.39091050119754	-2.22005815105577	2.91250515662490	
C	2.76630211309324	-2.99204300766795	3.30045750414760	
H	2.15088036925686	-1.28734352831236	2.19553169392071	
H	0.97703611467115	1.86144595161866	-4.06171040861175	
H	0.20443636065224	1.76602818447838	-2.47706024900179	
H	0.36838164453016	3.32304711133077	-3.28615649800866	
H	3.33114979035648	2.85125598958790	-4.26546748107522	
H	2.77931626514165	4.35542947131591	-3.54460009447413	
H	4.14303102578875	3.49467763107883	-2.83760936358857	
H	0.99355895390623	1.15511657098887	4.07564365741155	
H	0.29669468642389	2.67005573950099	3.50019080126225	
H	0.28801226282391	1.24541189517049	2.46027682737811	
H	3.26026673638600	2.24180041907095	4.53165056576366	
H	4.05826403955324	3.18545089036775	3.27244719562788	
H	2.60800521533726	3.80172859373944	4.05809492343897	
H	3.71968440101335	-2.34770647994371	-4.47921206054646	
H	4.34832125183044	-3.26945973762410	-3.11316769767576	
H	2.96586732207017	-3.86628374693832	-4.02585095491999	
H	1.43641005260843	-1.17405258206719	-4.28748178635071	
H	0.65970576255098	-2.66383748970025	-3.74887526927058	
H	0.56783979450821	-1.21528723151344	-2.75061992354013	
H	0.40357949255119	-1.66863702931007	3.85209749571540	
H	-0.27990014411254	-1.70518809917523	2.22722738722276	
H	-0.02602589741813	-3.20617151079596	3.11150862725613	
H	2.80257286480982	-2.45033420080533	4.24528084780641	
H	2.45634951380942	-4.01398850233586	3.51557061566717	
H	3.77565210677058	-3.02872224615517	2.89156601884081	

1a-VB (X =[Al($C_4N_2H_6O$)], L=PH_3)

(energies in Hartree)

C	4.27658302975178	-1.92764326700511	0.47002821874891
C	4.73518527352386	-1.00582514468629	-0.38559292646978
C	4.65911169206905	1.39412592622093	-0.08234718036499
O	3.95949374167083	0.19513806191485	-0.42653142023021
C	4.14640849689021	2.04758445993050	0.96751681697057
N	3.07644892337153	1.52449750556862	1.64700774894619
Al	2.38406389289779	-0.01919136925968	0.87059827418478
Au	0.08011102181491	-0.01339933712182	0.18014586663529
P	-2.30370760292321	0.00052435074266	-0.57239449275748
N	3.18487932948294	-1.65534753677180	1.25364373891158
H	5.56125460225917	-1.05566995224192	-1.07081105013591
H	4.75673407219450	-2.89752502384915	0.52374886368365
H	2.81079351865511	-2.44280989178502	1.75330625314038
H	5.47763638024820	1.66142772564719	-0.72496569076224
H	4.56512433755863	3.00218769182301	1.26336153112453
H	2.65752780228049	2.13897446465938	2.32275862437894
H	-2.84100258571679	-1.11863614827873	-1.24207416199447
H	-3.33429003550886	0.15677533552723	0.37778334322146
H	-2.74317392352030	0.98545975896511	-1.48132325523123

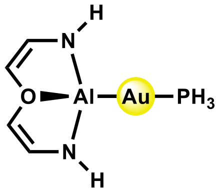

E_{SCF}(B97-3c)
= -1062.191325968701

E_{SCF}(PBE0/def2-TZVPP//B97-3c)
= -1061.691828588259

$E_{CASSCF(4,4)}$
= -1057.528803130500

Thermal correction* (B97-3c)
= 0.08648985

***1 atm, 298.15 K, 1M.**

2a-VB (X = [BH_2], L=PH_3)

(energies in Hartree)

B	2.25975224308406	-0.22105779886496	0.00000829716141
Au	0.22643976227576	-0.03459025734304	-0.00446533115460
P	-2.21779321482610	0.20319645586456	-0.00359714318173
H	2.91317678202194	-0.07968876355593	0.99809982041736
H	2.87992519843497	-0.47899782595556	-0.99599268648844
H	-2.96707933392959	-0.42390614333603	1.01044383967845
H	-2.96954398386737	-0.23044166749723	-1.11270710070580
H	-2.75745195019366	1.49826888968818	0.11555107427335

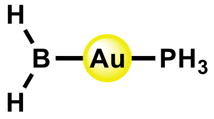

E_{SCF}(B97-3c)
= -505.162151777146

E_{SCF}(PBE0/def2-TZVPP//B97-3c)
= -504.781955676127

$E_{CASSCF(4,4)}$
= -502.980613909169

Thermal correction* (B97-3c)
= 0.01349285

***1 atm, 298.15 K, 1M.**

Me–Au–P^{t}Bu$_3$

(energies in Hartree)

Au	0.37910154920158	0.38066662502957	0.01758038467033
P	1.70541489274554	1.69413516539578	1.45299063809582
C	-0.79117457180441	-0.77599046069066	-1.24386497213926
H	-0.24006235730326	-1.05958844276317	-2.14205080323227
H	-1.11532482928676	-1.69269002152419	-0.74844811901997
H	-1.68421531226256	-0.23327027121481	-1.55824087337653
C	0.64980173526887	2.24240033243847	2.95692299911116
C	1.45097332273148	2.77472906932601	4.14071110130700
C	-0.20257686116280	1.05002902469314	3.40664763337714
H	-0.80869420029601	0.67076806018486	2.58716246376740
H	0.37802419192766	0.22793256666197	3.80255906055141
H	-0.87493004203545	1.38802087423384	4.19673628240028
H	2.07073103483211	2.00995375553770	4.59825288080403
H	0.75662869572635	3.12382221653338	4.90703945239525
H	2.08805440852669	3.61160661020049	3.87316558291858
C	3.19909035558362	0.65169765176311	2.05025124594221
C	3.72379425158691	-0.15709337124177	0.85847539448462
C	2.70786325752984	-0.38040608661472	3.06593187167490
H	2.42799271355529	0.05761703885752	4.01752679491206
H	1.86944262634619	-0.95532163013476	2.67971656977649
H	3.52208236967240	-1.07935971152851	3.26135750256109
H	2.93670123746643	-0.77322037769164	0.42988268393060
H	4.51588958305087	-0.81795986140773	1.21405688817768
H	4.34093290041038	2.52187957403738	-0.00991460245299
C	2.32767150125058	3.23592282889783	0.49907694320702

E_{SCF}(B97-3c)
= -990.598832733013

E_{SCF}(PBE0/def2-TZVPP//B97-3c)
= -990.012744884730

Thermal correction* (B97-3c)
= 0.35525351

***1 atm, 298.15 K, 1M.**

C	1.17145796399253	3.76689940212594	-0.35677924435036
C	3.41069638955853	2.80448259189298	-0.49011321576087
H	4.13839394601151	0.45342812153741	0.06765886361866
H	3.07497089923966	1.98520369625350	-1.12177295857394
H	3.63066878100421	3.65083535888034	-1.14203433297556
H	0.78914780941029	2.99407292698513	-1.01957307378453
H	1.54782730553532	4.58590676774801	-0.97166616845997
H	0.34322189570812	4.14919168625168	0.22447773675170
C	4.33781838105778	1.45730097383926	2.66761118450290
H	4.01339530682286	2.06391232661934	3.50702910695481
H	5.09752507049760	0.76656555877510	3.03782428460336
H	4.82399108027593	2.10840558577553	1.94789626370294
C	-0.34256919425964	3.31238263673928	2.50085024813094
H	0.12756733221282	4.26276799572582	2.27410400942811
H	-0.91580241032838	2.98550162521949	1.63633657065702
H	-1.04807116089789	3.48977452051800	3.31355120179589
C	2.87147970269604	4.35822215876004	1.37750533670366
H	2.10235122636032	4.80727843375670	1.99792905405809
H	3.68041459267665	4.03219753851333	2.02364745229647
H	3.26476879516415	5.14786675010399	0.73472723985661

Me–Au–IPr

IPr = *N*,*N'*-bis(dipp)imidazole-2-ylidene, dipp = 2,6-$^{i}Pr_2C_6H_3$.

(energies in Hartree)

C	3.78734169059468	-2.44849840503479	0.10645200017618
C	3.86551450326560	-1.19732691437217	-0.51010316902843
C	4.06141077228125	-1.03334589380340	-1.88359411127508
C	4.20200706264398	-2.18636480576849	-2.65066954107604
C	4.14273871013688	-3.44119711310109	-2.06753437717281
C	3.93441930109285	-3.57031334880271	-0.70440784449254
N	3.70886739965118	-0.02705147153238	0.30109065337414
C	4.74121953263890	0.64624895552812	0.93031662255913
C	4.17522774428758	1.68153144682337	1.59075217034854
N	2.81569410011085	1.60729548033833	1.34326726978531
C	2.50789356520310	0.55408852221339	0.54562153384537
Au	0.67012469021600	-0.02541072131992	-0.13568777295954
C	1.83751801178316	2.52164610945823	1.85326507488419
C	1.22845361226361	2.23087974434034	3.07665489728585
C	0.27316730202632	3.12894835044997	3.54392194581700
C	-0.05734995478831	4.25973485565765	2.81586683287267
C	0.55728348566496	4.51452076129425	1.60121994593359
C	1.51913214392017	3.64802275170493	1.09013425897217
C	1.54192747263519	0.97225324744950	3.85020804293110
C	1.98764289482931	1.27175272563862	5.27859868461079
C	2.14449882168380	3.90588178611022	-0.26035057968432
C	2.81127592511784	5.27661927061884	-0.33230988261956
C	4.07354025956491	0.32862597836096	-2.53660772792683
C	5.36536295556443	0.58004670447125	-3.30915776136486
C	3.50804809414673	-2.60090027690073	1.58305172971069
C	4.60249569850413	-3.39410978300656	2.29181435488604
C	0.35142752752146	0.01543527864009	3.81956773822453
C	1.11205268913717	3.72297049765384	-1.37137923877279
C	2.84122298626990	0.51084769763594	-3.42069905922734
C	2.12857377720888	-3.21673108106068	1.80943910852970
H	4.60079707055966	2.45441167884419	2.20277650278807
H	5.76293407840785	0.32641999539156	0.84772406740389
H	-0.22733834686138	2.93489624192894	4.48259569091562
H	-0.80547819306504	4.94374616696711	3.19416984512129
H	0.27596218400848	5.39274037942530	1.03651917723010
H	4.34679996670424	-2.10138984924021	-3.71890232200571
H	4.25019986136796	-4.32559888295092	-2.68149618550703
H	3.87252937649554	-4.55679815319501	-0.26594375142282
H	2.92195104241397	3.16119613416071	-0.41933038541845
H	2.36889131617703	0.46842566636330	3.35338990006183
H	4.01531757143025	1.08029934609798	-1.75171893153286
H	3.48940505493321	-1.60792275017294	2.02805208637060
H	2.81921642281411	1.51691642015271	-3.83878049946111
H	2.83947895596824	-0.19759549194364	-4.24836288771446
H	1.92875591033165	0.36148156749698	-2.84514959206695
H	5.37642960135702	1.59530589244615	-3.70453351797709
H	6.23886320600999	0.45609601776094	-2.66992017080964
H	5.46969693404270	-0.10173835979091	-4.15214817416423
H	4.41237320050459	-3.42302131241748	3.36430794427025
H	4.64576850928262	-4.42326799777087	1.93794251792274
H	5.58229655679667	-2.94502352102379	2.13309873873862
H	1.90216468675412	-3.25846393211532	2.87454460553427
H	1.35590355705685	-2.62603957214353	1.31964923829554
H	2.07950735726186	-4.23145630148392	1.41581762387124
H	2.27495062945138	0.34998697675010	5.78358869621691
H	2.84274515351145	1.94675358124674	5.29390147676526
H	1.19037967819512	1.72987444716296	5.86227944407779
H	0.60301418253188	-0.92041000892740	4.31806652426744
H	-0.51269990873024	0.44436663234796	4.32590400722695
H	0.06477147848050	-0.21152528199813	2.79364706097890

E_{SCF}(B97-3c)
= -1335.500171218346

E_{SCF}(PBE0/def2-TZVPP//B97-3c)
= -1334.743071377256

Thermal correction* (B97-3c)
= 0.53908188

***1 atm, 298.15 K, 1M.**

H	1.58006181319196	3.84034621908257	-2.34846069889383	
H	0.66514373428011	2.73111274939208	-1.32395728863915	
H	0.31303130527182	4.45907832116075	-1.28991090270009	
H	3.31599567008840	5.39885383995531	-1.29019591065330	
H	2.08550674824049	6.08334266350117	-0.23954422555761	
H	3.55087597326816	5.39995456926758	0.45811375779892	
C	-1.18670270429814	-0.61447451040351	-0.82382466835204	
H	-1.22641739567572	-0.62572764561857	-1.91539694871528	
H	-1.44135999338374	-1.61982663051237	-0.48090738885607	
H	-1.97536929435229	0.05670388412043	-0.47681869155535	

References

1. F. Neese, *WIREs: Comput. Mol. Sci.*, 2018, **8**, e1327.
2. F. Neese, *WIREs: Comput. Mol. Sci.*, 2012, **2**, 73-78.
3. J. G. Brandenburg, C. Bannwarth, A. Hansen and S. Grimme, *J. Chem. Phys.*, 2018, **148**, 064104.
4. A. D. Becke, *J. Chem. Phys.*, 1997, **107**, 8554-8560.
5. F. Weigend and R. Ahlrichs, *Phys. Chem. Chem. Phys.*, 2005, **7**, 3297-3305.
6. D. Andrae, U. Häußermann, M. Dolg, H. Stoll and H. Preuß, *Theor. Chim. Acta*, 1990, **77**, 123-141.
7. R. Flores-Moreno, R. J. Alvarez-Mendez, A. Vela and A. M. Köster, *J. Comput. Chem.*, 2006, **27**, 1009-1019.
8. S. Grimme, J. Antony, S. Ehrlich and H. Krieg, *J. Chem. Phys.*, 2010, **132**, 154104.
9. S. Grimme, A. Hansen, J. G. Brandenburg and C. Bannwarth, *Chem. Rev.*, 2016, **116**, 5105-5154.
10. V. Barone and M. Cossi, *J. Phys. Chem. A*, 1998, **102**, 1995-2001.
11. G. Knizia, http://www.iboview.org/.
12. G. Knizia, *J. Chem. Theory Comput.*, 2013, **9**, 4834-4843.
13. G. Knizia and J. E. M. N. Klein, *Angew. Chem. Int. Ed.*, 2015, **54**, 5518-5522.
14. C. Adamo, M. Cossi and V. Barone, *J. Mol. Struct.: THEOCHEM*, 1999, **493**, 145-157.
15. F. Neese, F. Wennmohs, A. Hansen and U. Becker, *Chem. Phys.*, 2009, **356**, 98-109.
16. K. Kitaura and K. Morokuma, *Int. J. Quantum Chem*, 1976, **10**, 325-340.
17. T. Ziegler and A. Rauk, *Inorg. Chem.*, 1979, **18**, 1755-1759.
18. T. Ziegler and A. Rauk, *Inorg. Chem.*, 1979, **18**, 1558-1565.
19. G. te Velde, F. M. Bickelhaupt, E. J. Baerends, C. Fonseca Guerra, S. J. A. van Gisbergen, J. G. Snijders and T. Ziegler, *J. Comput. Chem.*, 2001, **22**, 931-967.
20. E. Van Lenthe and E. J. Baerends, *J. Comput. Chem.*, 2003, **24**, 1142-1156.
21. E. V. Lenthe, E. J. Baerends and J. G. Snijders, *J. Chem. Phys.*, 1993, **99**, 4597-4610.
22. E. Van Lenthe, E. J. Baerends and J. G. Snijders, *J. Chem. Phys.*, 1994, **101**, 9783-9792.
23. E. Van Lenthe, J. G. Snijders and E. J. Baerends, *J. Chem. Phys.*, 1996, **105**, 6505-6516.
24. E. Van Lenthe, R. van Leeuwen, E. J. Baerends and J. G. Snijders, *Int. J. Quantum Chem*, 1996, **57**, 281-293.
25. E. Van Lenthe, A. Ehlers and E.-J. Baerends, *J. Chem. Phys.*, 1999, **110**, 8943-8953.
26. T. Lu and F. Chen, *J. Comput. Chem.*, 2012, **33**, 580-592.
27. T. Lu and F.-W. Chen, *Acta Physico-Chimica Sinica*, 2012, **28**, 1-18.
28. F. L. Hirshfeld, *Theor. Chim. Acta*, 1977, **44**, 129-138.
29. C. Fonseca Guerra, J.-W. Handgraaf, E. J. Baerends and F. M. Bickelhaupt, *J. Comput. Chem.*, 2004, **25**, 189-210.
30. R. S. Mulliken, *J. Chem. Phys.*, 1955, **23**, 1833-1840.
31. P. O. Löwdin, *J. Chem. Phys.*, 1950, **18**, 365-375.
32. A. D. Becke, *J. Chem. Phys.*, 1988, **88**, 2547-2553.
33. P. J. Stephens, F. J. Devlin, C. F. Chabalowski and M. J. Frisch, *J. Phys. Chem.*, 1994, **98**, 11623-11627.
34. A. D. Becke, *J. Chem. Phys.*, 1993, **98**, 5648-5652.
35. C. Lee, W. Yang and R. G. Parr, *Phys. Rev. B*, 1988, **37**, 785-789.

36. A. D. Becke, *Phys. Rev. A*, 1988, **38**, 3098-3100.
37. S. Grimme, S. Ehrlich and L. Goerigk, *J. Comput. Chem.*, 2011, **32**, 1456-1465.
38. H. Kruse and S. Grimme, *J. Chem. Phys.*, 2012, **136**, 154101.
39. S. Grimme, J. G. Brandenburg, C. Bannwarth and A. Hansen, *J. Chem. Phys.*, 2015, **143**, 054107.
40. E. Caldeweyher, S. Ehlert, A. Hansen, H. Neugebauer, S. Spicher, C. Bannwarth and S. Grimme, *J. Chem. Phys.*, 2019, **150**, 154122.
41. C. Bannwarth, S. Ehlert and S. Grimme, *J. Chem. Theory Comput.*, 2019, **15**, 1652-1671.
42. I. F. Leach, L. Belpassi, P. Belanzoni, R. W. A. Havenith and J. E. M. N. Klein, *ChemPhysChem*, 2021, **22**, 1262-1268.
43. I. F. Leach, R. W. A. Havenith and J. E. M. N. Klein, *Eur. J. Inorg. Chem. (under review)*, 2022.
44. J. H. Van Lenthe and G. G. Balint-Kurti, *Chem. Phys. Lett.*, 1980, **76**, 138-142.
45. J. H. v. Lenthe and G. G. Balint-Kurti, *J. Chem. Phys.*, 1983, **78**, 5699-5713.
46. J. H. van Lenthe, F. Dijkstra and R. W. Havenith, *Theor. Comput. Chem.*, 2002, **10**, 79.
47. J. Verbeek, J. H. Langenberg, C. P. Byrman, F. Dijkstra, R. W. A. Havenith, J. J. Engelberts, M. Zielinski, Z. Rashid and J. H. Van Lenthe, *TURTLE, an ab initio VB/VBSCF program*, Utrecht, The Netherlands, 1988.
48. M. F. Guest, I. J. Bush, H. J. J. Van Dam, P. Sherwood, J. M. H. Thomas, J. H. Van Lenthe, R. W. A. Havenith and J. Kendrick, *Mol. Phys.*, 2005, **103**, 719-747.
49. J. Pipek and P. G. Mezey, *J. Chem. Phys.*, 1989, **90**, 4916-4926.
50. G. Gallup and J. Norbeck, *Chem. Phys. Lett.*, 1973, **21**, 495-500.
51. B. H. Chirgwin and C. A. Coulson, *Proc. R. Soc. London, Ser. A*, 1950, **201**, 196-209.
